# Comparing Aerobic Interval Training with Other Forms of Physical Exercise for Brachial Artery Endothelial Function Improvement: A Systematic Review and Network Meta-analysis of Randomized Controlled Trials

**DOI:** 10.1186/s40798-025-00929-3

**Published:** 2025-11-21

**Authors:** Armin H. Paravlic, Simon Iskra, Ensar Abazovic, Nicola Lamberti, Fabio Manfredini, Kristina Drole

**Affiliations:** 1 Faculty of Sport, University of Ljubljana, Gortanova 22, 1000 Ljubljana, Slovenia; 2 Institute for Kinesiology Research, Scientific Research Center Koper, Koper, Slovenia; 3 Faculty of Sports Studies, Incubator of Kinanthropology Research, Masaryk University, Brno, Czechia; 4 Faculty of Sport and Physical Education, University of Sarajevo, Patriotske lige 41, 71000 Sarajevo, Bosnia and Herzegovina; 5 Department of Neuroscience and Rehabilitation, University of Ferrara, Ferrara, Italy; 6 Rehabilitation Medicine Unit, University Hospital of Ferrara, Ferrara, Italy

## Abstract

**Background:**

Brachial artery endothelial function, measured by the flow-mediated dilatation (FMD) technique, serves as a surrogate for coronary endothelial function and is recognized as an independent predictor of cardiovascular disease risk. Despite the known benefits of physical exercise interventions (PEI) in improving endothelial function, limited evidence exists to guide practitioners on the most effective form of PEI for enhancing endothelial function. The aim of this article is to investigate the effects of different PEI modalities on brachial artery FMD, and to establish the most effective PEI through a systematic review and network meta-analysis (NMA).

**Methods:**

PubMed, WoS, CINAHL, EMBASE, CENTRAL and EBSCOhost search was conducted from inception to February 20th, 2025. Randomized controlled studies investigating the effects of PEI on brachial artery FMD in adults were included. Both pairwise and Bayesian NMA were conducted using random-effects model to compare different PEI modalities within primary (aerobic training, resistance training and combined training) and secondary (continuous aerobic training vs. interval aerobic training vs. dynamic resistance training vs. combined training) categorizations. The PEI effectiveness was ranked using the surface under the cumulative ranking curve (SUCRA).

**Results:**

In total, 84 studies with 3596 participants (43% females, 51.9 ± 15.1 years of age) were included in the analysis. Summarized evidence of 119 effect sizes through pairwise comparisons showed improvement in FMD (mean difference [MD], 2.24%; 95% confidence interval [CI] 1.90–2.58, p < 0.001) following different PEI, without difference between magnitude of the effect between healthy and asymptomatic individuals (Q, 1.27, p = 0.260). As shown in the NMA, the rank order within a primary classification showed aerobic training as the most effective (SUCRA: 89.8%, MD, 2.37%, 95% credible interval [CrI] 1.95–2.80) followed by resistance training (SUCRA: 66.0%, MD, 2.07%, 95% CrI, 1.34–2.79), and combined (aerobic and resistance) training (SUCRA: 44.1%, MD, 1.67%, 95% CrI, 0.73–2.6). Secondary NMA identified interval aerobic training as the most effective (SUCRA: 99.1%, MD, 3.07%, 95% CrI, 1.37–3.76), which showed to be more effective than continuous aerobic training (MD, 1.08%), dynamic resistance training (MD, 1.04%), and combined training (MD, 1.36%). Moreover, a negative association was found between FMD improvement and both intervention duration and overall training load, while positive associations were observed with weekly training frequency, single session duration, and weekly training duration.

**Conclusions:**

Various PEI modalities have demonstrated effectiveness in improving brachial artery FMD, with interval aerobic exercises of higher intensities emerging as the most effective based on current evidence, followed by dynamic resistance training, continuous aerobic training and combined training. These findings have significant implications for informing future exercise guidelines aimed at both prevention and treatment of endothelial dysfunction.

The study protocol was prospectively registered in PROSPERO online registry: ID: CRD42023453202

**Supplementary Information:**

The online version contains supplementary material available at 10.1186/s40798-025-00929-3.

## Key points

• In this meta-analysis of 84 randomized controlled trials, physical exercise interventions (PEIs) in general significantly increased brachial artery endothelial function measured by flow-mediated dilatation (FMD) technique in the adult population.
• This large-scale systematic review and network meta-analysis showed that aerobic training, resistance training and combined training are all effective in enhancing brachial artery FMD.
• Although various PEIs have demonstrated effectiveness in enhancing brachial artery FMD, interval aerobic exercises of higher intensities are emerging as the most effective based on current evidence.
• The findings from network meta-regression analyses suggest that greater improvements following PEI can be achieved by increasing the duration of acute stimuli (an additional half-hour of training = 0.80% increase in FMD) and weekly training exposure (an additional two and a half hours of training on weekly basis = 0.50% increase in FMD).

**Supplementary Information:**

The online version contains supplementary material available at 10.1186/s40798-025-00929-3.

## Background

Non-communicable diseases are a major global public health challenge, of which cardiovascular disease (CVD) remains the world’s leading cause of morbidity and mortality [1]. CVD is generally manifested through diseases of heart and blood vessels, often leading to chronic conditions and events such as heart attacks, stroke, heart failure, and peripheral artery disease. It is estimated that CVD itself accounts for approximately 17.9 million of deaths annually [2]. Given its substantial societal burden, several world-leading health organizations are called upon to take action by seeking potential solutions for the prevention and management of major CVD risk factors [3–5].

Traditional risk factors for CVD include older age, smoking, hypertension, being overweight or obese, diabetes, high cholesterol, and a family history of heart disease [6, 7]. In recent years, several other i.e., non-traditional risk factors have been identified, among which vascular function assessed by means of flow-mediated dilatation (FMD) response of the brachial artery represents an important marker of vascular health [8]. FMD is an index of endothelial-dependent vasodilation, with larger dilatory responses reflecting increased endothelial function [8]. Conventionally, brachial endothelial function is recognized as a surrogate for coronary endothelial function [9] and an independent predictor of CVD risk [10, 11]. A meta-analysis of 14 studies including 5547 subjects showed that a 1% decrease in brachial artery FMD is associated with an 8% increase in the risk of future cardiovascular events [10]. Thus, for both primary and secondary prevention of CVD, it would be beneficial to improve endothelial health through subject’s tailored interventions.

Physical exercise interventions (PEI) have long been recognized as effective measures in both the prevention and management of several noncommunicable diseases [12–17]. These interventions have been shown to positively impact various physiological markers, including lowering cholesterol [18], glucose levels [19], and blood pressure [20], thus reducing the risk of CVD, diabetes, and metabolic syndrome. In light of this, a hypothesis has emerged suggesting that PEI may improve endothelial function measured by FMD, which is a key indicator of vascular health [21–24].

Recent systematic reviews highlight the efficacy of PEI in improving FMD across diverse populations [21, 25–28]. Nonetheless, high methodological heterogeneity was observed in the published literature, particularly regarding participant’s characteristics, the types of PEI implemented, intervention durations, and other factors related to the interventions themselves and FMD assessment protocols [21, 25–28]. For example, a meta-analysis of 8 studies (n = 208) by Sabouri and colleagues [21] showed that high-intensity interval training enhanced FMD by + 1.8% compared to moderate-intensity interval training in overweight/obese adults. Another meta-analysis found a 3.1% of increase in FMD in favor of aerobic training compared to non-intervention control group in patients with heart failure with reduced ejection fraction [26]. A recent umbrella review summarized findings from 19 meta-analysis investigating the optimal training regimen for improving FMD [25]. The evidence suggests that the effectiveness of PEI depends on the health status of the participants involved. For example, healthy individuals may benefit the most from higher intensity aerobic exercise and resistance training of low to moderate intensity. Similarly, CVD patients may benefit the most from high intensity aerobic exercise, whereas patients with type 2 diabetes were shown to have the most benefits from low intensity PEI [25]. Notably, heart failure patients exhibited + 1.5% FMD gains with supervised aerobic regimens (3 × /week, 60–90 min/session) [26], and Campbell and colleagues emphasized sustained FMD improvements (+ 1.6%) in older adults (≥ 60 years) engaged in long-term aerobic training [27]. Additionally, Son and colleagues corroborated PEI’s efficacy in overweight/obese adults (+ 1.9% FMD), particularly with ≥ 12-week programs [28]. However, despite the established efficacy of PEI in enhancing endothelial function being once again confirmed, a notable gap persists in the existing literature to inform clinicians and practitioners with quantitative evidence on determining the most beneficial form of PEI for improving endothelial function. This gap can be addressed by implementing network meta-analysis (NMA) [29, 30]. The advantage of NMA over other meta-analytic approaches lies in its ability to provide quantitative evidence for both direct and indirect comparisons of various PEIs that have not been directly investigated in original studies [30]. To the best of the author’s knowledge, there is no published NMA aimed at investigating the effectiveness of PEI on endothelial function.

Therefore, considering the large number of original studies that have been conducted, the primary aim of this article is to combine direct and indirect evidence of randomized controlled studies investigating the effectiveness of PEI on brachial artery endothelial function measured by FMD through the NMA approach.

## Methods

### Eligibility Criteria, Literature Search and Study Selection

This review study has been conducted in accordance with the Preferred Reporting Items for Systematic reviews and Meta-Analysis (PRISMA) statement [31] and its extension statement for Reporting of Systematic Reviews Incorporating Network Meta-analyses of Health Care Interventions [30]. The protocol was prospectively registered in the PROSPERO online registry (ID: CRD42023453202).

The systematic search of PubMed, Web of Science, CINAHL, EMBASE, the Cochrane Central Register of Controlled Trials and EBSCOhost (including MEDLINE, Science Citation Index Expanded, Scopus, SPORTDiscus, DOAJ and ERIC) was conducted from inception to 20th of February, 2025. The following terms and their combinations were used as a search string: adult, vascular endothelium, endothelial function, endothelial dysfunction, flow-mediated dilatation, endothelium-dependent vasodilatation, vascular reactivity, exercise, physical exercise, exercise training, randomized controlled trial. Furthermore, the combination of relevant medical subject heading (MeSH) terms with the Boolean operators “OR”, “AND” and “NOT” was used if possible. In addition, the reference list from the retrieved articles as well as those from the systematic literature reviews and meta-analysis where hand searched for additional eligible articles. For detailed search strategy refer to Supplementary file, Sect. 1.

Reports were deemed eligible if they were appropriately randomized, reported pre- and post-intervention FMD (%) values in both the exercise intervention group and the non-intervention control group, and considered the intervention, comparator, and outcome as defined below. To reduce the confounding factors, any studies that reported additional interventions (e.g., counseling/education, caloric restriction, exercise) in the non-intervention control group were excluded. Studies that combined other interventions with exercise (such as the use of supplements or medication modifications, behavioral changes consoling, blood flow restriction, hypoxic environment etc.) were also excluded. Dissertation theses were not eligible since only trials that were published in peer-reviewed publications were taken into consideration. Eligibility criteria were selected in accordance with the PICOS approach. *Population*: studies recruiting adult subjects (≥ 18 years of age), with no restriction to sex, ethnicity or health status; *Intervention*: PEI with an eligible non-intervention control group; PEI was defined as any physical activity that increases energy expenditure and involves planned or structured body movements performed systematically and designed to maintain or enhance health-related outcomes. *Comparison*: Change in FMD was compared across various modes of physical exercise interventions using our primary and secondary categorization (see Supplementary file, Sect. 2). PEI included in the primary categorization were aerobic training (AT), resistance training (RT) and combined training (CT = AE + RT). For clarity in classifying PEI in this manuscript, we used the following definitions: AT refers to PEI that engages large muscle groups, can be maintained continuously and is rhythmic in nature (e.g., cycling, running); RT is a form of PEI aimed at improving muscular fitness by exercising a muscles or a muscle groups against external resistance (e.g., weight lifting, squats, body weight exercises, etc.,); and CT integrates both AT and RT. The secondary categorization included continuous AT (CAT), interval AT (IAT), dynamic RT (DRT), and CT. *Outcome*: brachial artery endothelial function measured by the FMD technique; *Study design*: randomized controlled trials (RCTs).

### Screening Strategy and Data Extraction

**Fig. 1:**
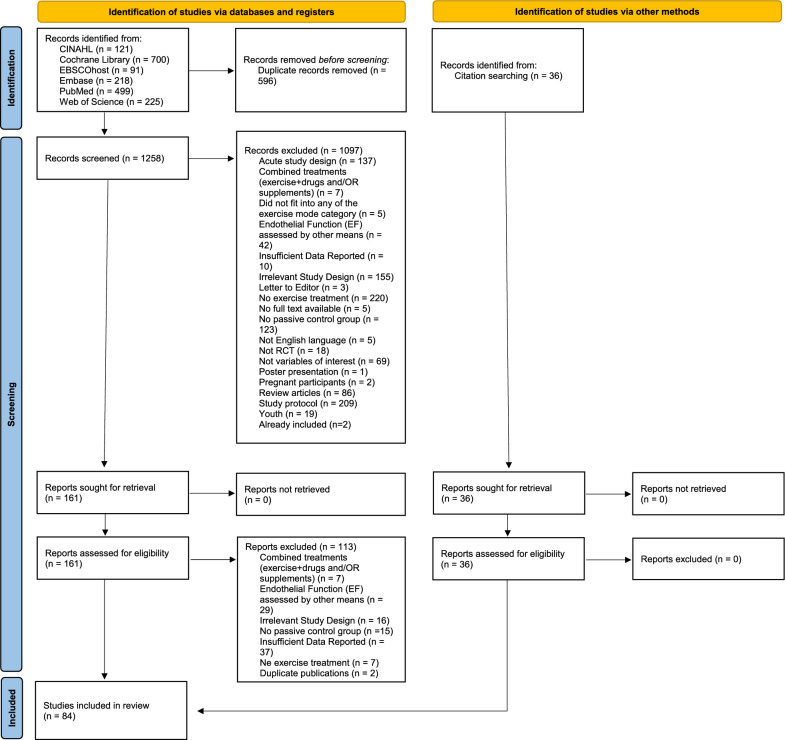
PRISMA flow diagram of study selection process

Literature search and identification of the studies was performed by two authors (AHP and NL), whereas screening of the articles for eligibility was performed in pairs by 6 reviewers (AHP, EA, SI, KD, FM, NL) independently. During the preliminary phases, an online platform Nested Knowledge (Saint Paul, Minnesota, USA) was used [32]. In the first step, all references were uploaded to the Nested Knowledge platform, where automatic screening for duplicates was performed. Secondly, titles and abstracts were assessed using predetermined eligibility criteria stated above. Thirdly, the full-text articles of the remaining reports that met the preliminary inclusion criteria were retrieved and reviewed by three reviewers to reach a final decision for inclusion in the synthesis. Any disagreements between reviewers were resolved by consensus or consultation with the leading author (AHP), if needed. In case the full-text was not available online, the corresponding author was contacted by e-mail or through the ResearchGate platform. The entire process of the study selection process is presented in Fig. 1. Criteria for data extraction were discussed and accepted by the authors. Data extraction according to the predefined criteria was undertaken by four team members in pairs and checked by EA independently. Any uncertainties were discussed with leading author until the agreement was reached.

### Methodological Quality Assessment

Methodological quality of the included studies was assessed using the Physiotherapy Evidence Database (PEDro) scale. The PEDro scores ≥ 3, 4 to 5 and from 6 to 10, were categorized as poor quality, fair quality and high quality studies, respectively [33]. In addition, a revised Cochrane risk-of-bias tool for randomized trials was used to assess risk of bias in included studies [34].

### Credibility Assessment

To assess the credibility of each comparison against control group, a Confidence in NMA (CINeMA) approach was used [35]. In line with the recommended guidelines, judgements were made for several factors including within-study bias, reporting bias, indirectness, imprecision, heterogeneity, and incoherence, for each comparison [35]. Similar to Grading of Recommendations Assessment, Development and Evaluation approach, the evidence for comparisons was initially considered to show high confidence, then downgraded based on concerns in each domain, as follows:

*Within study bias*—Comparisons were downgraded when most of the studies providing direct evidence for comparisons were evaluated as high risk.

*Reporting bias*—Publication bias was assessed by ROB-MEN tool, which is a web-application tool for the assessment of the risk of bias due to missing evidence in NMA [36].

*Indirectness***—**Our primary population of interest were adults, regardless of their health status. Studies were considered to be indirect if they focused on one sex only (> 90% male or female), participants with diagnosed diseases (i.e., symptomatic) or older adults (> 60 years of age). We marked the study as showing “some concerns” if one of these factors was present, and as “major concerns” if two or more of these factors were present.

*Imprecision***—**As per CINeMA, we used the clinically important difference to establish a zone of equivalence, where differences were not considered clinically relevant. Thus, a threshold of 1% of brachial artery FMD improvement was chosen, as it showed to be clinically relevant [11]. Studies were automatically marked as “some concerns” for imprecision if the bounds of the 95% credible interval extended across that zone, and they were marked as “major concerns” if the bounds extended to the other side of the zone of equivalence.

*Heterogeneity***—**CINeMA accounts for heterogeneity by assessing whether the prediction intervals and the credible intervals lead to different conclusions about the clinical significance (using the same zone of equivalence from imprecision). Comparisons are marked as “some concerns” if the prediction interval crosses into, or out of the zone of equivalence once (e.g., from helpful to no meaningful effect), and as “major concerns” if the prediction interval crosses the zone twice (e.g., from helpful and harmful).

*Incoherence***—**Incoherence assesses whether the NMA provides similar estimates when using direct evidence (e.g., randomized controlled trials on AT vs. RT) compared with indirect evidence (e.g., randomized controlled trials where either AT or RT uses waitlist control). Incoherence provides some evidence the network may violate the assumption of transitivity: that the only systematic difference between arms is the treatment, not other confounders. In current study, the incoherence was assessed using a global design-by-treatment interaction to assess for incoherence across the whole network. The comparisons were marked as “some concerns” if either no direct comparisons were available or direct and indirect evidence gave different conclusions about clinical significance (e.g., from helpful to no meaningful effect, as per imprecision and heterogeneity). Again, the comparisons were classified as “major concerns” if the direct and indirect evidence changed the sign of the effect or changed both limits of the credible interval.

### Statistical Analysis

The pairwise meta-analyses were performed by the MASimplified online tool [37]. The MASimplified tool is powered by Rstudio and Shiny. All Bayesian statistical calculations are performed using R package *metaphor* [38]. Due to large methodological and statistical heterogeneity observed within the included studies and for each meta-analysis, data were analysed using a random effect model. Egger’s test was performed on the collected data to provide statistical evidence of publication bias (p < 0.10) [39]. As FMD of the brachial artery was measured by standardized procedures using an ultrasound imaging technique and reported as percentage, mean difference (MD) with 95% confidence intervals (CI) were calculated. The MD was calculated by subtracting the mean change in the comparison group (i.e. control group) from the mean change in the reference group (i.e. AT, RT, CT etc.,). In case of Bayesian analysis, a MD with 95% credible interval (CrI) was calculated and reported accordingly. Heterogeneity was assessed with I^2^ statistic that indicates the percentage of variability across studies due to heterogeneity rather than chance. Values of 25%, 50% and 75% represent low, moderate and high heterogeneity. Several pairwise subgroup meta-analyses were performed to compare primary PEI categories (AT vs RT vs CT); secondary categories (CAT vs. IAT vs DRT vs. CT); participant’s health status (healthy vs. symptomatic); and training duration (≤ 4 weeks, 5–12 weeks, 13–24 weeks, and ≥ 25 weeks). Meta-analysis was performed only if three or more analysis units were included for the specific endpoint. A level of p ≤ 0.05 was adopted as statistically significant for all analyses performed.

To investigate the effectiveness of PEI that have not been directly compared in original investigations, the Bayesian network meta-analyses (NMAs) were performed by the MetaInsight tool (version v6.3.0) [40]. The MetaInsight tool is powered by Rstudio and Shiny. All Bayesian statistical calculations are performed using R package *gemtc* [41] and R package BUGSNET [42]. For all NMAs, random-effects analyses were selected for the same reasons as for pairwise meta-analyses. Inconsistency between direct and indirect effect comparisons were investigated by node-splitting models [41]. Further, a sensitivity analyses were performed by excluding studies with large residual deviance (≥ 2) from the model. To investigate the effectiveness of different PEIs, a separate NMAs were conducted for primary and secondary PEI categories (for details please refer to Comparison within a 2.1 section). For better visualisation of direct and indirect comparisons, a network graphical illustration was used [43]. Moreover, a probability ranking analyses were conducted, wherein surface under the cumulative ranking curve (SUCRA) values were computed for each NMAs. These values were then graphically presented as litmus rank-o-gram SUCRA plots [43]. In addition, a network meta-regression analyses were performed to investigate whether the effects of PEI on brachial artery FMD were moderated by initial FMD of the participants, their age, and body mass index (BMI), intervention duration, weekly training frequency, single session duration, weekly training duration and overall training load (intervention duration in weeks * weekly training frequency * single session duration in minutes).

## Results

### Study Selection Process

The performed search of six databases (PubMed, Web of Science, CINAHL, EMBASE, the Cochrane Central Register of Controlled Trials and EBSCOhost) yielded 1854 reports, of which 596 duplicates were excluded. In total, 1258 reports were then screened and 1097 were excluded for reasons highlighted in the Fig. 1. Among the remaining 161 reports, all reports were assessed for eligibility, whereas 113 were excluded for one or more of the following reasons: (a) report combined several treatments (n = 7), (b) endothelial function was assessed by other means (n = 29), (c) not RCTs (n = 16), (d) no passive control group (n = 15), (e) insufficient data reported to be included in the meta-analysis (n = 37), (f) No exercise treatment / other exercise treatments of interest (n = 7), (g) duplicated publication (n = 2). Finally, 84 articles (36 records identified through citation searching) were eligible for inclusion.

### Study and Participant’s Characteristics

**Fig. 2:**
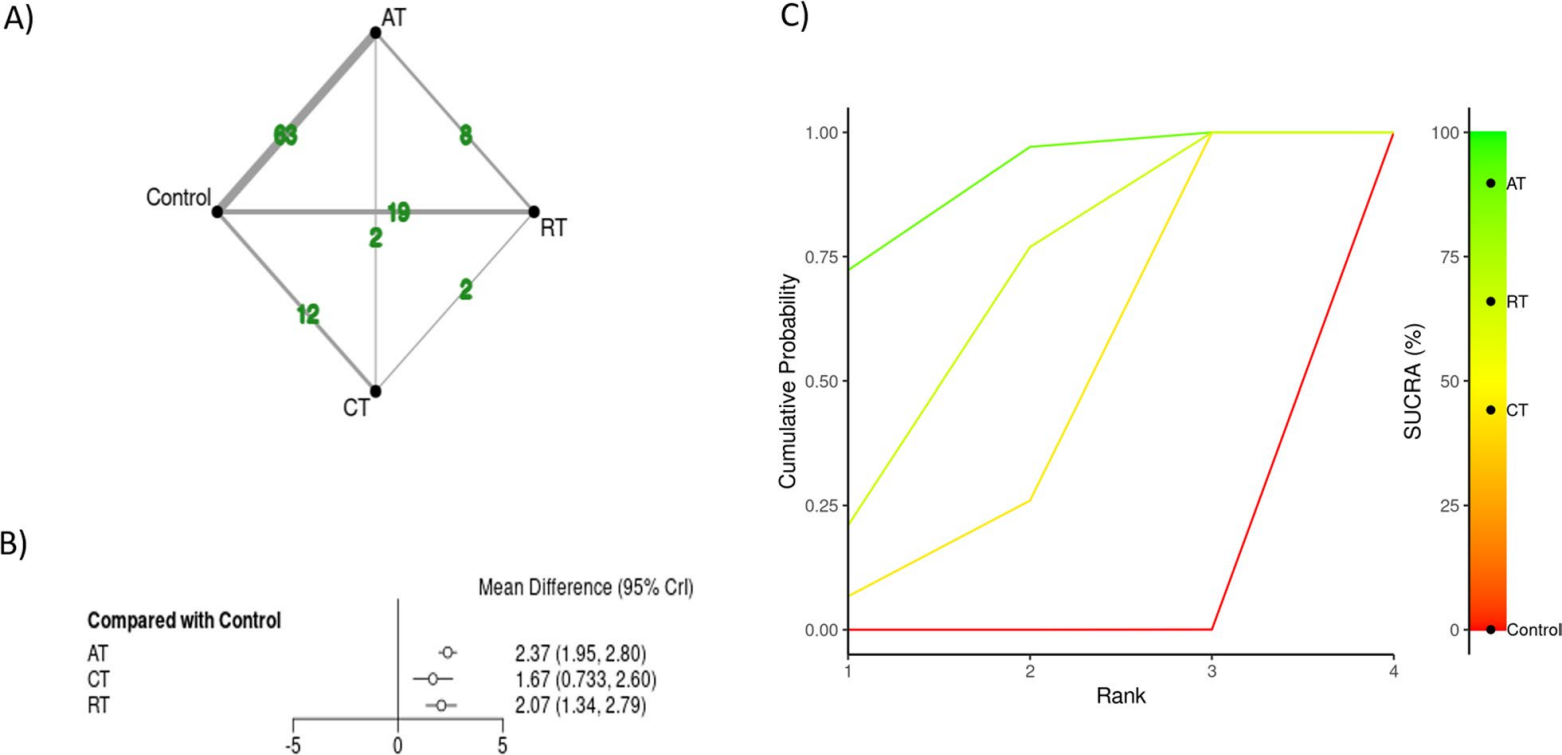
Network diagrams depicting the direct and indirect comparisons for the primary network meta-analyses (**A**), with corresponding forest plot (**B**), and Bayesian ranking panel plots (**C**). *AT* aerobic training, *RT* resistance training, *CT* combined training (CT = AE + RT), *SUCRA* surface under the cumulative ranking curve. Results were reported as mean differences with 95% credible intervals

**Fig. 3:**
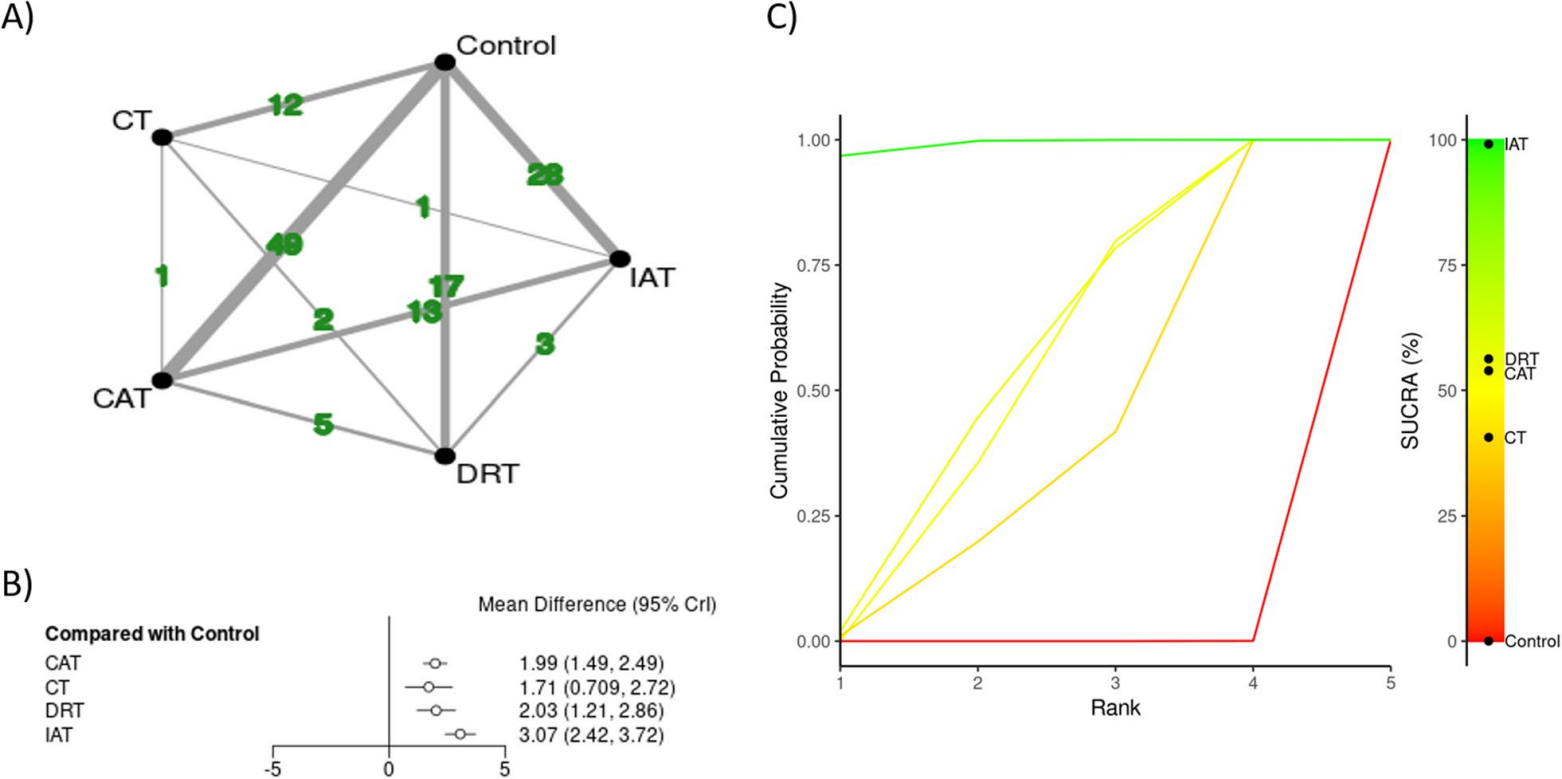
Network diagrams depicting the direct and indirect comparisons for the secondary network meta-analyses (**A**), with corresponding forest plot (**B**), and Bayesian ranking panel plots (**C**). *CAT* continuous aerobic training (AT), IAT interval AT, *DRT* dynamic resistance training (RT), *CT* combined training (CT = AE + RT), SUCRA surface under the cumulative ranking curve. Results were reported as mean differences with 95% credible intervals

In total, 84 studies with 3596 participants (43% females, 51.9 ± 15.1 years of age on average, range: 19–78 years) were included in current review. In total, 2284 participants were recruited in the experimental groups with an average of 19 participants per group (range, 5–93 per group). Within a primary categorization, studies included 63 AT [23, 24, 44–106], 19 RT [23, 45, 49–53, 63, 80, 107–117], and 12 CT [45, 53, 114, 118–123] PEI interventions (Fig. 2A). A secondary categorization yielded 49 CAT, 28 IAT, 17 DRT, 12 CT PEIs (Fig. 3A). Study duration ranged from 4 to 52 weeks (11.7 weeks on average).

A systematic overview of the studies included in the meta-analysis with their main characteristics and results is presented in Supplementary Table 4, Sect.  3. For sensitivity and comparative analyses, we conducted primary pairwise meta-analyses and NMA analyses in parallel, excluding studies investigating PEI effectiveness on FMD in symptomatic population such as patients diagnosed with cardiovascular diseases, metabolic disorders, neurological or mental problems (Supplementary Table 4, Sect.  3). It is noteworthy that the inclusion or exclusion of such diseases does not substantially impact the overall results. Thus, the results were interpreted on the whole sample.

### Methodological Quality Assessment of the Individual Studies

**Fig. 4:**
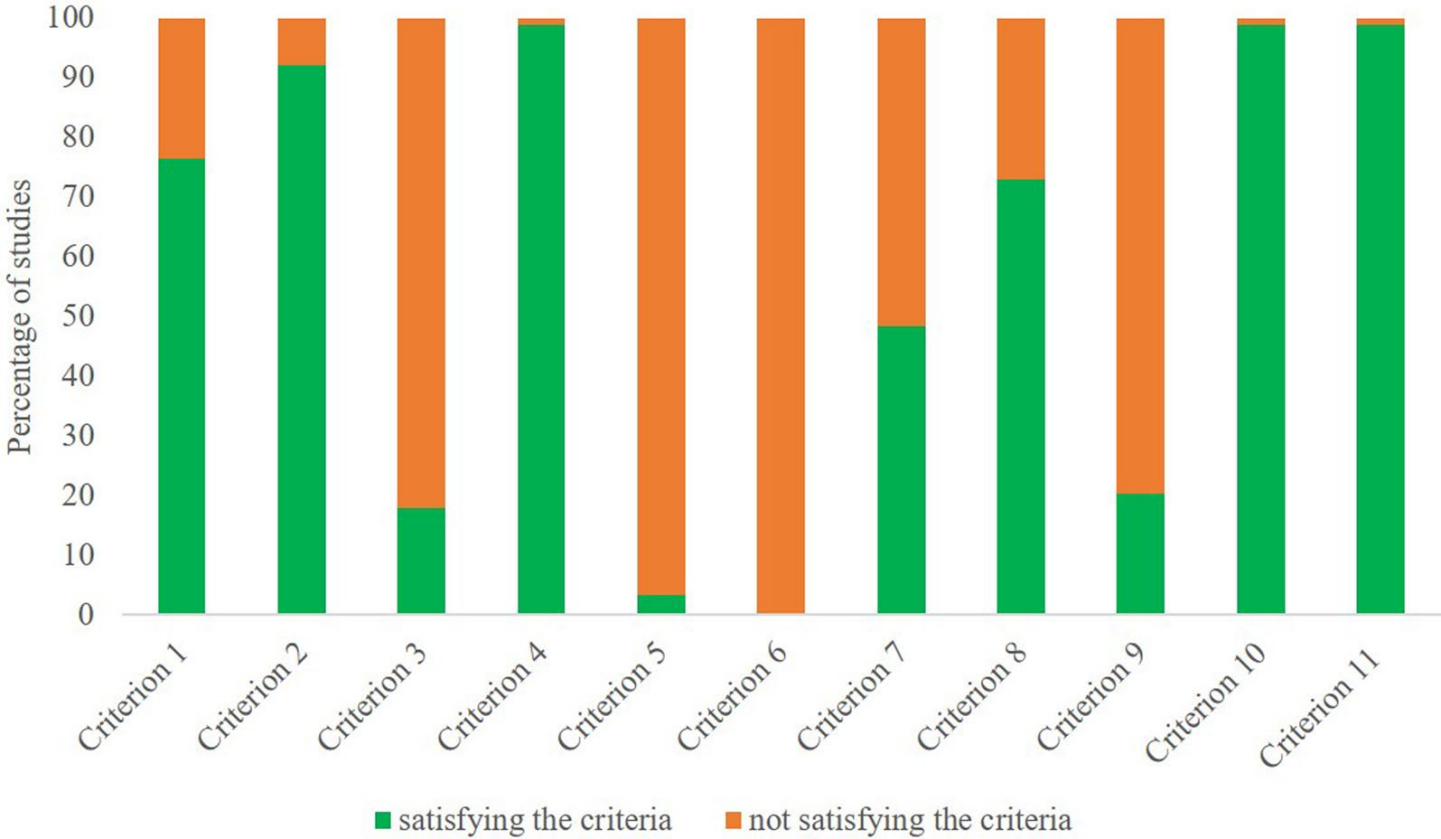
Fulfillment of physiotherapy evidence database (PEDro) criteria for the studies included in the present meta-analysis

Methodological quality of the included studies showed that included studies were on average of fair quality with an average PEDro score of 5.1 (range from 3–8). Most of the studies failed to report whether the allocation to group was concealed (82%), blinding of the subjects (97%), blinding of the therapist (100%), and whether the subjects for whom outcome measures were available received the treatment or control condition as allocated (80%) (Fig. 4). Based on predetermined thresholds, 47% and 51% of studies were evaluated as having low and moderate risk of bias, whereas only 2% of studies were evaluated as having high risk of bias (Fig. 4). In contrary, the majority of studies failed to satisfy criteria 3, 5 and, 6, which were related to concealed allocation, blinding of all subjects and all therapists that administered the intervention. Here, we emphasize that blinding participants is challenging to achieve in studies using physical exercise interventions as a treatment. For more details about each study PEDro score please refer to Supplementary Table 5, Sect.  4.

### Pairwise Meta-analysis

#### Effects of Physical Exercise Intervention on Endothelial Function Measured by FMD Technique

A summarized evidence included 119 effect sizes (ES) showing improvement in FMD (MD, 2.24%; 95% CI 1.90–2.58, 95% PI, −0.85–5.32, p < 0.001) following different PEI (Supplementary Table 6, Sect.  5). Sub-group analysis showed that both healthy (MD, 1.94%; 95% CI 1.32–2.56, p < 0.001) and symptomatic FMD (MD, 2.36%; 95% CI 1.95–2.77, p < 0.001) individuals improved FMD following PEI in general, without difference between groups (Q, 1.27, p = 0.260) (Supplementary Table 6, Sect.  5). Results of Egger’s test indicated publication bias for overall effect (p < 0.001).

A summarized evidence for primary classification showed improvement following all PEI ranging from 1.65% (CT, p < 0.001) to 2.36% (AT, p < 0.001), without difference between groups (Q, 1.85, p = 0.396).

A meta-analysis for secondary classification showed that all PEIs significantly improved FMD, whereas IAT showed to be the most beneficial (MD, 2.91%; 95% CI 2.01–3.81, p < 0.001) (Supplementary Table 8, Sect. 5). There was no difference between different PEIs (Q, 3.86, p = 0.277).

### Network Meta-analyses

#### Credibility Assessment

The results of CINeMA are reported in the Supplementary file, Sect.  6 for all three classification categories of PEIs. Results varied between very low to high ratings of confidence for primary and secondary classification.

#### The Primary Physical Exercise Intervention Classification

The results from NMA considering primary PEI categorization are presented in Fig. 2 through network graphical illustration (Fig. 2A), forest plot (Fig. 2B), and ranking probabilities (Fig. 2C). The primary PEI NMA on FMD included 84 original studies (3596 participants), of which 76 were two-arm studies and 8 were identified as multi-arm studies. The order of effectiveness based on SUCRA values showed AT (SUCRA: 89.8%) as the most effective PEI, followed by RT (66.0%), and CT (44.2%) All treatments, have shown to be effective in improving FMD when compared to control, however, no significant differences between treatments were observed (Table 1). There was no evidence of inconsistency in the primary NMA (all p ≥ 0.159).

**Table 1:** Comparative network meta-analysis for brachial artery endothelial function considering primary physical exercise intervention classification

	AT	Control	CT	RT
AT	AT	− 2.37 (− 2.8, − 1.95)	− 0.71 (− 1.72, 0.29)	− 0.3 (− 1.1, 0.48)
Control	**2.37 (1.95, 2.8)**	Control	**1.67 (0.73, 2.6)**	**2.07 (1.34, 2.79)**
CT	0.71 (− 0.29, 1.72)	− 1.67 (− 2.6, − 0.73)	CT	0.4 (− 0.74, 1.55)
RT	0.3 (− 0.48, 1.1)	− 2.07 (− 2.79, − 1.34)	− 0.4 (− 1.55, 0.74)	RT

Bolded values represent a statistically significant result

*AT* aerobic training, *RT* resistance training, *CT* combined training (CT = AE + RT)

A sensitivity analysis was run by excluding four studies [24, 83, 94, 97] because of high residual deviance, without compromising results of a preliminary analysis.

#### The Secondary Physical Exercise Intervention Classification

The results from NMA considering secondary PEI categorization are presented in Fig. 3 through network graphical illustration (Fig. 3A), forest plot (Fig. 3B), and ranking probabilities (Fig. 3C). Other supporting material can be found in Supplementary File, Sect.  7. The secondary NMA included 82 original studies (3497 participants), of which 62 were two-arm studies and 21 were identified as multi-arm studies. In this analysis, two studies investigating isometric resistance training were excluded [109, 116]. All treatments, showed to be effective in improving FMD when compared to control. The order of effectiveness based on SUCRA values showed IAT (SUCRA: 99.1%) as the most effective PEI, followed by DRT (59.3%), CAT (53.9%), and CT (40.7%). Moreover, only IAT showed to be significantly more effective in improving FMD when compared to other PEIs such as CAT (MD, 1.08%, 95% CrI, 0.35–1.81), CT (MD, 1.36%, 95% CrI, 0.18–2.54), and DRT (MD, 1.04%, 95% CrI, 0.04–2.05) (Table 2).

**Table 2:** Comparative network meta-analysis for brachial artery endothelial function considering secondary physical exercise intervention classification

	CAT	Control	CT	DRT	IAT
CAT	CAT	− 1.99 (− 2.49, − 1.49)	− 0.28 (− 1.38, 0.83)	0.04 (− 0.88, 0.96)	**1.08 (0.35, 1.81)**
Control	**1.99 (1.49, 2.49)**	Control	**1.71 (0.71, 2.72)**	**2.03 (1.21, 2.86)**	**3.07 (2.42, 3.72)**
CT	0.28 (− 0.83, 1.38)	− 1.71 (− 2.72, − 0.71)	CT	0.32 (− 0.93, 1.57)	**1.36 (0.18, 2.54)**
DRT	− 0.04 (− 0.96, 0.88)	− 2.03 (− 2.86, − 1.21)	− 0.32 (− 1.57, 0.93)	DRT	**1.04 (0.04, 2.05)**
IAT	− 1.08 (− 1.81, − 0.35)	− 3.07 (− 3.72, − 2.42)	− 1.36 (− 2.54, − 0.18)	− 1.04 (− 2.05, − 0.04)	IAT

*CAT* continuous aerobic training (AT), *IAT* interval AT, *DRT* dynamic resistance training (RT) and *CT* combined training (CT = AE + RT)

The inconsistency between direct and indirect evidence was observed in the secondary NMA comparing CAT and IAT (p = 0.03). This inconsistency persisted even after excluding five studies [24, 78, 83, 94, 97] due to high residual deviance. Despite this variability, the network estimate (integrating direct and indirect evidence) retained a significant overall effect (MD: −0.743; 95% CrI: -1.46, −0.0327), reinforcing directional consistency across methodologies.

#### Network Meta-regression Analyses

The results of the network meta-regression analyses are presented in Table 3. The findings demonstrate that the effect of PEI on participant’s brachial FMD was moderated by initial brachial FMD (shared mean β = 0.26, 95% CrI 0.20–0.32), and BMI (β =− 0.13, 95% CrI − 0.20 to − 0.07), with a negative association observed, suggestingthe lower the initial BMI, the larger the effect of PEI. Moreover, all training-related variables of interest showed significant moderating effects on FMD, but with varying directions. For example, negative associations were observed between intervention duration (β = -0.54, 95% CrI − 0.59 to − 0.48) and overall training load (β = − 0.42, 95% CrI − 0.49 to − 0.36) with FMD improvement. In contrast, positive associations were found between FMD improvement and weekly training frequency (β = 0.27, 95% CrI 0.21–0.33), single session duration (β = 0.80, 95% CrI 0.72–0.88), and weekly training duration (β = 0.50, 95% CrI 0.43–0.57), respectively. No significant associations were observed for the participants age.

**Table 3:** Model fit summaries for univariate network meta-regression

Covariate	DIC	pD	Residual deviance	Shared beta (mean)	95% CrI		SD	Number of data points	Mean value	Scale number
					Lower limit	Upper limit				
Brachial FMD at baseline	340.69	160.08	180.61	0.26	0.204	0.323	0.4067	178	5.99	4.12
Participant’s age	341.26	160.12	181.14	0.04	− 0.020	0.105	0.4237	178	51.93	30.43
Participant’s BMI	294.53	138.60	155.93	− 0.13	− 0.195	− 0.065	0.4127	154	27.96	5.86
Intervention duration (weeks)	337.88	157.60	180.28	− 0.54	− 0.595	− 0.484	0.3755	176	11.56	9.35
Weekly training frequency	333.69	156.62	177.07	0.27	0.209	0.331	0.4133	174	3.29	2.19
Single session duration (min)	295.99	139.15	156.84	0.80	0.732	0.877	0.4434	142	43.31	27.31
Weekly training duration (min)	298.08	140.32	157.76	0.50	0.428	0.574	0.4636	155	154.90	159.14
Overall training load (min)	294.43	138.65	155.78	− 0.42	− 0.492	− 0.357	0.4248	153	1692.87	1975.04

*CrI* credible interval, *BMI* body mass index, *DIC* deviance information criterion, *pD* probability of direction, *SD* standard deviation

## Discussion

In the current systematic literature review with NMA, a total of 84 relevant randomized controlled studies with 3596 participants were included and subsequently analyzed to investigate the effectiveness of different PEI modalities on brachial artery endothelial function measured by FMD. Both pairwise and NMAs demonstrated a significant improvement of FMD in the intervention groups compared to non-intervention controls; however, the magnitude of their effectiveness substantially varied. As shown by NMA for primary PEI classification, the rank order of PEI effectiveness based on SUCRA values showed AT as the most effective PEI, followed by RT and CT, and that all PEI modalities significantly improved FMD compared to controls (Fig. 2). NMA on secondary PEI classification found that all treatments were effective in improving FMD when compared to controls. The order of effectiveness based on SUCRA values showed IAT as the most effective PEI, followed by DRT, CAT, and CT. Moreover, when considering direct evidence, only IAT showed to be significantly more effective in improving FMD compared to other PEIs such as CAT, CT and DRT. Finally, through network meta-regression analyses we demonstrated that participants brachial FMD and BMI at baseline, intervention duration, weekly training frequency, single session duration, weekly training duration and overall training duration moderated PEI effectiveness on FMD.

To the best of author’s knowledge, this is the first study aimed at summarizing the evidence on the effectiveness of different PEI modalities on brachial artery endothelial function measured by FMD through NMA. The most recent study investigated this topic through the umbrella review, and identified 27 systematic literature reviews, of which 19 were meta-analyses [25]. The authors concluded that in general, PEI is beneficial approach for improving FMD [25]. In particular, Shivgulam and colleagues [25] included studies evaluating PEI such as AT, RT, CT, Tai Chi and blood-flow restricted training. The latter study [25] authors found that healthy adults can benefit the most from higher intensity AT or low intensity resistance training, which is partly in line with our findings. The present study is the first to incorporate other subcategories of PEI that are directly and indirectly compared through secondary NMAs such as IAT, CAT, DRT, and CT, to identify the most effective PEI modalities considering several important domains of the exercise. Thus, in contrast to previous findings [25], we found that adults, regardless of their health status can benefit the most from IAT which is by nature of higher intensity compared to CAT for example. The positive association between AT intensity and improvement in FMD was previously established, which may be attributed to the greater release of nitric oxide caused by a greater shear stress on the endothelium resulting from higher exercise intensity [124, 125]. These mechanisms align with the emerging evidence from the present study, particularly when summarizing the evidence from secondary category of PEIs. NMAs within secondary category demonstrated that IAT is significantly more effective in improving FMD compared to CAT, DRT and CT, supported by a synthesis of direct, indirect, and network evidence. Although the effect magnitude differs between direct and indirect comparisons of IAT and CAT, the directional consistency (both favouring IAT), combined with statistically significant direct evidence and supportive indirect trends, supports the conclusion that IAT elicits greater FMD improvements. Despite this variability, the network estimate (integrating direct and indirect evidence) retained a significant overall effect (MD: −0.743; 95% CrI: −1.46, −0.0327), reinforcing directional consistency across methodologies. Mechanistically, IAT’s intermittent high-intensity phases induce repeated, transient increases in shear stress and endothelial shear rate variability, which are potent stimuli for endothelial nitric oxide synthase activation and vascular adaptation [126]. In contrast, CAT’s sustained moderate-intensity shear stress may elicit a less pronounced adaptive response, consistent with prior evidence that pulsatile hemodynamic stimuli (as seen in interval training) optimize endothelial function [126, 127]. While the direct evidence strongly supports IAT’s superiority, the marginal inconsistency (node-split p = 0.0316) highlights the need for standardized protocols (e.g., harmonizing interval durations or intensity thresholds) in future trials.

While our findings support the effectiveness of IAT as a high-intensity exercise intervention among symptomatic populations, it is not routinely adopted in clinical practice. This hesitancy stems from persistent safety concerns among clinicians and researchers, particularly regarding cardiovascular risks. High-intensity exercise may pose a potential risks for patients with cardiovascular conditions due to acute hemodynamic stress it places on the heart and vasculature, potentially triggering adverse events such as arrhythmias, myocardial ischemia, or acute cardiovascular events such as myocardial infarction [128, 129]. Therefore, further implementation studies in controlled environments are essential to fully understand the safety and efficacy of high-intensity exercise interventions tailored to patients with underlying health conditions affecting the heart and cardiovascular system. Our findings indicate that both healthy and symptomatic adults can benefit from IAT, CAT, DRT, and CT in improving endothelial health. Practitioners have the flexibility to select from various exercise modalities based on the goal of improving endothelial health, particularly in asymptomatic individuals.

It is important to note that different exercise modalities primarily target different fitness capacities: resistance training is most effective for improving muscular strength, aerobic training offers greater benefits for cardiovascular fitness, and combined training provides a balance between both [3]. The superior improvements in brachial FMD following IAT compared to RT or CT may stem from its unique hemodynamic and endothelial stimulus [130, 131]. IAT’s intermittent high-intensity phases generate repetitive, pulsatile increases in shear stress—while recovery periods allow for transient ischemia–reperfusion cycles that further enhance vascular adaptation. In contrast, resistance training predominantly induces acute, pressure-dominated hemodynamic loads (e.g., elevated blood pressure during lifting), which may transiently impair endothelial function or fail to elicit sustained shear-mediated vasodilation [132]. Combined training, while integrating aerobic and resistance elements, may dilute the shear stress magnitude or frequency required for optimal endothelial adaptation, as resistance components could counteract the shear-mediated benefits of aerobic exercise. Additionally, IAT’s structured high-intensity intervals may more effectively upregulate antioxidant defenses and reduce oxidative stress, mitigating endothelial dysfunction in a manner not replicated by resistance or CT protocols [127, 133]. These findings align with evidence that shear stress patterns, rather than exercise volume or muscle hypertrophy, are critical determinants of FMD improvement, underscoring the specificity of vascular adaptations to exercise modality. Therefore, when prescribing exercise, practitioners should prioritize the modality based on the individual patient’s needs and preferences. However, given the moderate to large heterogeneity of the observed effects, exercise prescriptions should be individualized and guided by fundamental training principles [134].

The findings from the network meta-regression analysis provide valuable insights into how PEIs influence vascular function, and how these effects are moderated by factors such as patient’s initial brachial FMD, BMI, and all training-related variables, but with varying directions. A negative association between baseline BMI and FMD improvement (shared mean β = −0.13) suggests that individuals with lower BMI at the baseline tend to experience greater improvements in FMD following PEI. This observation aligns with previous research indicating that excess adipose tissue can negatively impact endothelial function by promoting a pro-inflammatory state that reduces nitric oxide bioavailability, which is essential for vasodilation [135–138]. Consequently, overweight or obese individuals may require more tailored or multi-modal interventions that combine exercise with dietary changes to maximize vascular health improvements [139]. Considering training characteristics, negative associations were observed between intervention duration and overall training load with FMD improvement (β = −0.54 and β = −0.42, respectively). These findings indicate that longer interventions and higher training volumes do not necessarily produce greater vascular benefits, a counterintuitive result may be explained by two interconnected mechanisms. First, prolonged training may lead to diminishing returns in vascular function, where physiological adaptations plateau or even regress due to factors such as overtraining, inadequate recovery, or oxidative stress that may develop over time [140]. Second, short-term interventions generate acute, repeated shear stress stimuli that enhance endothelial function through nitric oxide-mediated vasodilation [126]. In contrast, prolonged training promotes structural vascular remodeling (e.g., arterial lumen enlargement), which normalizes resting shear stress and blunts relative FMD% over time despite preserved vasodilatory capacity [141]. This temporal dynamic was corroborated by pairwise subgroup meta-analysis, which revealed the largest FMD improvements in programs ≤ 4 weeks (MD = 4.28%), with progressively smaller effects in longer interventions (e.g., 25 + weeks: MD = 1.77%). These results underscore the importance of balancing intervention duration to maximize early functional gains driven by endothelial adaptation before structural changes obscure FMD% improvements. On the contrary, positive associations were found between FMD improvement and weekly training frequency (β = 0.27), single session duration (β = 0.80), and weekly training duration (β = 0.50). These findings are in agreement with previous studies examining a similar question in general population [127] and in patients with heart failure [16, 26]. Ashor and colleagues found a positive association between resistance training frequency and improvements in FMD [127], whereas Fuertes-Kenneally and colleagues [26] found that more than two sessions are necessary to improve systemic endothelial function, as measured by brachial artery FMD in non-exercising muscles, in patients with heart failure. However, we observed additional benefits from single session duration and total weekly training duration, adding new insights that suggest greater vascular improvements can be achieved by focusing on the regularity and structure of training sessions, rather than the overall duration of the intervention. Contrary to previous findings [142], which reported a negative association between baseline brachial FMD and its improvement following PEIs (β = −0.47), we observed a significant positive association in our cohort. This divergence may reflect differences in study populations and methodological contexts. A positive association suggests that individuals with better baseline endothelial function retain greater vascular plasticity, enabling enhanced adaptive responses to exercise. Conversely, the negative association reported in [142] could indicate a floor effect, where populations with severe baseline endothelial dysfunction (e.g., advanced cardiovascular disease or diabetes) exhibit limited capacity for improvement due to pre-existing vascular damage.

Notably, no significant associations were observed between participant’s age and the degree of FMD improvement, suggesting that PEIs can be broadly effective across different age groups. This highlights the potential of exercise interventions as a universal tool for improving cardiovascular health, regardless of an individual’s starting point [25, 26, 138]. Overall, these findings underscore the importance of a balanced, regular exercise routine that prioritizes frequency and session duration over extended or overly intense training programs.

### Clinical Implications

This meta-analysis has several important clinical implications. First, we found that exercise improves brachial artery FMD regardless of individual health status, suggesting PEI can be considered as an effective strategy for both the primary and secondary prevention. Second, although many PEIs were found to be effective in improving brachial artery FMD, several modalities showed to be more beneficial then others (favouring IAT over, CAT, DRT and CT), thus practitioners can choose these PEI modalities when seeking for the most effective treatment among many. Moreover, the meta-regression analysis identified several significant moderators of PEI effectiveness, including lower baseline BMI, higher FMD, shorter intervention duration, higher weekly training frequency, longer single session duration, greater weekly training duration, and lower overall training load. In general, a negative association was found between FMD improvement and both intervention duration and overall training load, while positive associations were observed with weekly training frequency, single session duration, and weekly training duration. These findings suggest that greater improvements following PEI can be achieved by increasing the duration of acute stimuli (an additional half-hour of training = 0.80% increase in FMD) and weekly training exposure (an additional two and a half hours of training on weekly basis = 0.50% increase in FMD). In contrast, longer interventions do not necessarily guarantee greater chronic improvements in FMD.

### Research Gaps Identified Through the Literature and Future Research Suggestions

Along with identifying the most effective PEI, a comprehensive screening of the existing literature enabled identification of some gaps regarding the type of PEIs administered in original studies and the populations studied. As expected, AT was overrepresented compared to other PEIs such as RT or CT (Fig. 2A). Similarly, high and very high intensities of exercise were underrepresented compared to moderate intensity exercise.

Additionally, healthy, asymptomatic individuals were underrepresented (36% of all included studies) compared to symptomatic individuals (64%), with more than half of the latter group consisting of patients diagnosed with some form of CVD. Therefore, future studies should focus on exploring these interventions in healthy individuals, as well as in populations at high cardiovascular risk—those associated with higher mortality rates and lower quality of life, such as patients diagnosed with knee or hip osteoarthritis, cancer, and/or metabolic syndrome.

### Strengths and Limitations

Lastly, our study has several limitations that have to be acknowledged. Firstly, we introduced several inclusion criteria for this review, which limits our ability to generalize the findings to combined interventions (e.g., exercise plus diet or education on healthy life style) or other markers of endothelial function beyond brachial artery FMD. While this can be viewed as a limitation, it can also be considered a strength, as we narrowed our focus to specific settings, minimizing the influence of other moderating factors that would be difficult to interpret. Secondly, the included studies varied in duration, ranging from 8 to 52 weeks (with only one study extending beyond 26 weeks, i.e., at 52 weeks [108]), and showed substantial differences in training frequency, session duration, overall training duration, and the equipment used to conduct the interventions. Thirdly, the heterogeneity of pooled populations (e.g., healthy individuals, patients with CVD/diabetes) may have influenced results by introducing confounding baseline differences in endothelial function or comorbidity-driven responses. Though sensitivity analyses suggested no significant subgroup differences, limited statistical power precluded robust stratification, potentially masking population-specific effects. Fourthly, the heterogeneity of pooled populations (e.g., healthy individuals, patients with CVD/diabetes) may have influenced results by introducing confounding baseline differences in endothelial function or comorbidity-driven responses. Furthermore, the included studies demonstrated moderate methodological quality, with evidence certainty ranging from very low to moderate for most analyses. Although only few studies explicitly followed established FMD assessment guidelines [8], most provided adequate methodological descriptions, including supine positioning (commonly used), a 5-min ischemic stimulus, and partial reporting of glyceryl trinitrate administration (applied in < 50% of studies) and blood velocity detection (~ 50% of studies). These factors partially alleviate—but do not resolve—concerns that protocol variability might bias observed outcomes. The persistent lack of adherence to standardized guidelines remains a critical limitation. Future research must rigorously adopt consensus-driven FMD protocols to strengthen methodological consistency, minimize variability, and improve interpretability, particularly given inconsistent reporting of pivotal parameters across existing literature. And lastly, a potential limitation of our analysis is the absence of allometric scaling in the majority of included studies. Scaling FMD% to account for baseline arterial diameter as recommended [146] minimizes confounding by vessel size and improves comparability across populations. The inability to normalize FMD% for baseline diameter may introduce bias, particularly when comparing cohorts with differing baseline vascular dimensions (e.g., age, sex, or health status). Future studies should prioritize reporting scaled FMD metrics to enhance validity and reduce heterogeneity in pooled analyses. Therefore, we believe these findings should be interpreted within the context of short-to-moderate-term intervention studies, and research implementing longer intervention periods is warranted to investigate long term benefits of PEI on FMD.

## Conclusions

Various PEI modalities have demonstrated effectiveness in enhancing brachial artery FMD when compared to non-intervention controls, with interval aerobic exercises of higher intensities emerging as the most effective based on current evidence. Moreover, the meta-regression analysis identified several significant moderators of PEI effectiveness, including lower baseline BMI, higher baseline FMD, shorter intervention duration, higher weekly training frequency, longer single session duration, greater weekly training duration, and lower overall training load. These findings have significant implications for informing future exercise guidelines aimed at both the prevention and treatment of endothelial dysfunction.

## Supplementary Information

Supplementary material 1.

## Data Availability

The datasets used and/or analyses during the current study are available from the corresponding authors on reasonable request.

## Abbreviations

FMD: Flow-mediated dilatation
PEI: Physical exercise interventions
SUCRA: Surface under the cumulative ranking curve
NMA: Network meta-analysis
CVD: Cardiovascular disease
REML: Restricted maximum likelihood
CI: Confidence interval
CrI: Credible interval
PEDro: Physiotherapy evidence database
CINeMA: Confidence in NMA
AT: Aerobic training
RT: Resistance training
CT: Combined training (CT = AE + RT)
CAT: Continuous AT
IAT: Interval AT
DRT: Dynamic RT
