# Supplementary material for: Comparing Aerobic Interval Training with Other Forms of Physical Exercise for Brachial Artery Endothelial Function Improvement: A Systematic Review and Network Meta-analysis of Randomized Controlled Trials
Source: Sports Med Open. 2025 Nov 21;11:133. doi: 10.1186/s40798-025-00929-3 (PMC12638609; doi:10.1186/s40798-025-00929-3)
Supplement: Supplementary file 1 — Supplementary material 1. [file 40798_2025_929_MOESM1_ESM.docx]

**Title:** **Comparing Aerobic Interval Training with Other Forms of Physical Exercise for Brachial Artery Endothelial Function Improvement: A Systematic Review and Network Meta-Analysis of Randomized Controlled Trials**

**Running title: Physical exercise and endothelial function**

Authors: Armin H. Paravlic^1,2,3^, Simon Iskra^1^, Ensar Abazovic^4^, Nicola Lamberti^5^, Fabio Manfredini^5,6^, Kristina Drole^1^

^1^Faculty of Sport, University of Ljubljana, Ljubljana, Slovenia

^2^Science and Research Centre Koper, Institute for Kinesiology Research, Koper, Slovenia

^3^Faculty of Sports Studies, Incubator of Kinanthropology Research, Masaryk University, Brno, Czechia

^4^Faculty of Sport and Physical Education, University of Sarajevo, Patriotske lige 41, Sarajevo 71000, Bosnia and Herzegovina

^5^Department of Neuroscience and Rehabilitation, University of Ferrara, Ferrara, Italy

^6^Rehabilitation Medicine Unit, University Hospital of Ferrara, Ferrara, Italy

**Corresponding author:*

Armin H. Paravlic

Faculty of Sport, University of Ljubljana

Gortanova 22, 1000 Ljubljana, Slovenia

E-mail: armin.paravlic@fsp.uni-lj.si

**E-mail; ORCID:**

Armin H. Paravlic: [armin.paravlic@fsp.uni-lj.si](mailto:armin.paravlic@fsp.uni-lj.si); 0000-0002-7748-8097;

***Supplementary File***

**Effects of physical exercise on endothelial function: a systematic review and network meta-analysis of randomized controlled trials**

**Contents**

[S1 – Search strategy 4](#_Toc196168331)

[Cumulative Index to Nursing and Allied Health Literature (CINAHL) 4](#_Toc196168332)

[Cochrane Central Register of Controlled Trials (CENTRAL) 4](#_Toc196168333)

[EBSCOhost 4](#_Toc196168334)

[EMBASE 4](#_Toc196168335)

[PubMed 5](#_Toc196168336)

[Web of Science 5](#_Toc196168337)

[S2 –Methodology used for categorization of physical exercise interventions 6](#_Toc196168338)

[Supplementary table 1. Characteristics of exercise (REF) 6](#_Toc196168339)

[Supplementary table 2. Indices of exercise intensity for endurance exercise from maximal exercise testing and training zones (REF) 6](#_Toc196168340)

[Supplementary table 3. Indices of exercise intensity for resistance training from a single maximal repetition (1RM) or maximal voluntary isometric contraction (MViC) (REF) and (REF) 7](#_Toc196168341)

[S3 – Characteristics of included studies 8](#_Toc196168342)

[Supplementary table 4. Characteristics of included studies 8](#_Toc196168343)

[S4 - Methodological quality assessment of individual studies 20](#_Toc196168344)

[Supplementary table 5. Fulfilment of Physiotherapy Evidence Database (PEDro) criteria for each of the studies included in the present meta-analysis 20](#_Toc196168345)

[S5 - Pairwise comparisons 24](#_Toc196168346)

[Supplementary table 6. Differences between healthy and symptomatic group in changes in endothelial function measured by flow-mediated dilatation technique considering following PEI in general (pairwise meta-analysis). 24](#_Toc196168347)

[Supplementary table 7. Changes in endothelial function measured by flow-mediated dilatation technique considering a primary physical exercise intervention classification (pairwise meta-analysis). 24](#_Toc196168348)

[Supplementary table 8. Changes in endothelial function measured by flow-mediated dilatation technique considering a secondary physical exercise intervention classification (pairwise meta-analysis). 25](#_Toc196168349)

[Supplementary table 9. Changes in endothelial function measured by flow-mediated dilatation technique considering a intervention duration (pairwise meta-analysis). 26](#_Toc196168350)

[S6 - Credibility assessment 27](#_Toc196168351)

[Supplementary table 10. Level of concern for each of the six domains for each comparison in the Network Meta-analysis within primary classification of PEI 27](#_Toc196168352)

[27](#_Toc196168353)

[References 28](#_Toc196168354)

# S1 – Search strategy

## Cumulative Index to Nursing and Allied Health Literature (CINAHL)

“endothelial function” AND ( exercise or physical activity or fitness or aerobic training or strength training ) AND ( randomized controlled trials or rtc or randomised control trials)

## Cochrane Central Register of Controlled Trials (CENTRAL)

("endothelial function index" OR "flow-mediated dilation" OR "vascular endothelium") AND ("exercise" OR "Physical activity") AND randomized controlled trial in All Text - (Word variations have been searched)

## EBSCOhost

AB ( (adult) AND (vascular endothelium OR endothelial function OR endothelial dysfunction OR endothelium-dependent vasodilatation OR vascular reactivity OR flow-mediated dilatation OR FMD) AND (exercise OR physical exercise OR training) ) AND TX ( randomised controlled trial or randomized controlled trial or rct )

## EMBASE

adult/exp AND (exercise/exp OR 'physical activity'/exp) AND (('vascular endothelium'/exp OR 'blood vessel endothelium' OR 'endangium' OR 'endothelium, blood vessel' OR 'endothelium, vascular' OR 'endovasculum' OR 'vascular endothelium') OR 'endothelial function'/exp OR ('flow-mediated dilation test'/exp OR 'FMD technique' OR 'FMD test' OR 'flow-mediated dilatation assessment' OR 'flow-mediated dilatation technique' OR 'flow-mediated dilatation test' OR 'flow-mediated dilation assessment' OR 'flow-mediated dilation technique' OR 'flow-mediated dilation test') OR 'endothelium dependent vasodilation'/exp OR 'endothelium dependent relaxation'/exp OR 'endothelium dependent vasodilatation'/exp) AND ('randomized controlled trial'/exp OR 'controlled trial, randomized' OR 'randomised controlled study' OR 'randomised controlled trial' OR 'randomized controlled study' OR 'randomized controlled trial' OR 'trial, randomized controlled')

## PubMed

| Search: **((adult[MeSH Terms]) OR (adult[Title/Abstract])) AND ((vascular endothelium[MeSH Terms]) OR (vascular endothelium[Title/Abstract]) OR (endothelial function[Title/Abstract]) OR (endothelial dysfunction[Title/Abstract]) OR (flow-mediated dilatation[Title/Abstract]) OR (endothelium-dependent vasodilatation[Title/Abstract]) OR (vascular reactivity[Title/Abstract])) AND ((exercise[MeSH Terms]) OR (exercise[Title/Abstract]) OR (physical exercise[MeSH Terms]) OR (physical exercise[Title/Abstract]) OR (exercise training[MeSH Terms]) OR (exercise training[Title/Abstract])) AND (randomized controlled trial[Publication Type])**  ("adult"[MeSH Terms] OR "adult"[Title/Abstract]) AND ("endothelium, vascular"[MeSH Terms] OR "vascular endothelium"[Title/Abstract] OR "endothelial function"[Title/Abstract] OR "endothelial dysfunction"[Title/Abstract] OR "flow mediated dilatation"[Title/Abstract] OR "endothelium dependent vasodilatation"[Title/Abstract] OR "vascular reactivity"[Title/Abstract]) AND ("exercise"[MeSH Terms] OR "exercise"[Title/Abstract] OR "exercise"[MeSH Terms] OR "physical exercise"[Title/Abstract] OR "exercise"[MeSH Terms] OR "exercise training"[Title/Abstract]) AND "randomized controlled trial"[Publication Type]  **Translations**  **adult[MeSH Terms]:** "adult"[MeSH Terms]  **vascular endothelium[MeSH Terms]:** "endothelium, vascular"[MeSH Terms]  **exercise[MeSH Terms]:** "exercise"[MeSH Terms]  **physical exercise[MeSH Terms]:** "exercise"[MeSH Terms]  **exercise training[MeSH Terms]:** "exercise"[MeSH Terms] |
| --- |

## Web of Science

**ALL=((adult) AND (vascular endothelium OR endothelial function OR endothelial dysfunction OR endothelium-dependent vasodilatation OR vascular reactivity OR flow-mediated dilatation OR FMD) AND (exercise OR physical exercise OR training) AND (randomized controlled trial))**

<https://www.webofscience.com/wos/woscc/summary/1a3773f4-3c37-4337-a0f4-006a12b1ba5d-a22695c4/relevance/1>

# S2 –Methodology used for categorization of physical exercise interventions

## Supplementary table 1. Characteristics of exercise [(REF)](https://www.escardio.org/Guidelines/Clinical-Practice-Guidelines/sports-cardiology-and-exercise-in-patients-with-cardiovascular-disease)

| Mode of exercise training | Subcategories | Primary classification | Secondary classification |
| --- | --- | --- | --- |
| Metabolic | Aerobic | 1. Aerobic training (AT) | - Continuous (CAT) - Interval (IAT) |
|  | Anaerobic | 1. Resistance training (RT) | - Dynamic RT (DRT) |
| Muscular work | Isometric - isotonic | 1. Combined training (CT = AE + RT) | - Combined training (CT) |
|  | Dynamic (concentric, eccentric) |  |  |
|  | Continuous vs interval |  |  |
| Type of exercise |  |  |  |
|  | Endurance - Aerobic |  |  |
|  | Strength or resistance training |  |  |
|  | Speed and speed endurance |  |  |
|  |  |  |  |
|  | Flexibility |  |  |
|  | Coordination and balance |  |  |

## Supplementary table 2. Indices of exercise intensity for endurance exercise from maximal exercise testing and training zones [(REF)](https://www.escardio.org/Guidelines/Clinical-Practice-Guidelines/sports-cardiology-and-exercise-in-patients-with-cardiovascular-disease)

| Exercise intensity | VO_2max_ (%) | HR_max_ (%) | HRR (%) | RPE scale | Training Zone |
| --- | --- | --- | --- | --- | --- |
| Low | <40 | <55 | <40 | 10-11 | Aerobic |
| Moderate | 40-69 | 55-74 | 40-69 | 12-13 | Aerobic |
| High | 70-85 | 75-90 | 70-85 | 14-16 | Aerobic + lactate |
| Very high | >85 | >90 | >85 | 17-19 | Aerobic + lactate + anaerobic |

## Supplementary table 3. Indices of exercise intensity for resistance training from a single maximal repetition (1RM) or maximal voluntary isometric contraction (MViC) [(REF)](https://www.sciencedirect.com/science/article/pii/S2095254615000678) and [(REF)](https://journals.lww.com/nsca-jscr/abstract/2004/05000/monitoring_exercise_intensity_during_resistance.27.aspx)

| Relative intensity | 1RM (%) | RPE descriptor | RPE scale | Our classification? |
| --- | --- | --- | --- | --- |
| Very heavy | 100 | Maximal | 10 | Heavy |
| Heavy | 90-95 | Very Hard | 7-9 |  |
| Moderately heavy | 85-90 | Hard | 5-6 | Moderate |
| Moderate | 80-85 | Somewhat Hard | 4 |  |
| Moderately light | 75-80 | Moderate | 3 |  |
| Light | 70-75 | Easy | 2 | Light |
| Very light | 65-70 | Very, Very Easy | 1 |  |
| Rest | - | Rest | 0 |  |

# S3 – Characteristics of included studies

## Supplementary table 4. Characteristics of included studies

| Reference | Gender | GBD Classification | Female (%) | Age (years) | EXP Sample size | Con Sample size | Sport discipline/training mode | Duration (weeks) | Training frequency (tpw) | SS duration (min) | CAT1 | CAT2 | EXP ∆ _ba_FMD% | CON ∆ _ba_FMD |
| --- | --- | --- | --- | --- | --- | --- | --- | --- | --- | --- | --- | --- | --- | --- |
| Abdi 2021 [1] | Women | Diabetes_kidney | 100 | 20-44 | 15 | 15 | treadmill running | 12 | 3 | 40 | AT | IAT | 6.4 | 0.1 |
| Aispuru-Lanche 2024 | Both | Cardiovascular | 17 | 58 | 28 | 24 | Cycling or treadmill running | 16 | 2 | 20 | AT | IAT | 2.6 | 0.5 |
| Aispuru-Lanche 2024 | Both | Cardiovascular | 19 | 57.9 | 28 | 24 | Cycling or treadmill running | 16 | 2 | 30 | AT | IAT | 5.6 | 0.5 |
| Almenning 2015 | Women | Healthy | 100 | 27.9 | 8 | 9 | resistance training | 10 | 3 | 35 | RT | DRT | 0.4 | -1.2 |
| Almenning 2015 | Women | Healthy | 100 | 26.3 | 8 | 9 | walk/run/cycle | 10 | 3 | 40 | AT | IAT | 2 | -1.2 |
| Alvarez 2024 | NR | Healthy | NR | 40 | 10 | 10 | Cycling, resistance training | 6 | 3 | 25 | CT | CT | 8.4 | 2.1 |
| Alvarez 2024 | NR | Cardiovascular | NR | 41.3 | 8 | 10 | Cycling, resistance training | 6 | 3 | 25 | CT | CT | 2.5 | -3.1 |
| Alvarez 2024 | NR | Cardiovascular | NR | 44.7 | 10 | 10 | Cycling, resistance training | 6 | 3 | 25 | CT | CT | 7.7 | -2.3 |
| Azadpour 2017 | Women | Healthy | 100 | 57.1 | 12 | 12 | treadmill walking or jogging | 10 | 3 | 32.5 | AT | CAT | 5.2 | -0.5 |
| Banks 2024 | Both | Cardiovascular | 62 | 53.5 | 13 | 13 | Resistance training | 9 | 3 | 40 | RT | DRT | 2.4 | 0.1 |
| Beck 2013 | Both | Healthy | 27 | 21.4 | 15 | 15 | resistance training | 8 | 3 | 60 | RT | DRT | 2.1 | -0.4 |
| Beck 2013 | Both | Healthy | 31 | 20.9 | 13 | 15 | treadmill | 8 | 3 | 60 | AT | CAT | 3.7 | -0.4 |
| Belardinelli 2005 | Men | Cardiovascular | 0 | 56.9 | 30 | 29 | cycling | 8 | 3 | 40 | AT | CAT | 2.8 | -0.2 |
| Belardinelli 2006 | Men | Cardiovascular | 0 | 54.2 | 15 | 12 | cycling | 8 | 3 | 40 | AT | CAT | 2.3 | 0 |
| Belardinelli 2006 | Men | Cardiovascular | 0 | 54.3 | 15 | 10 | cycling | 8 | 3 | 40 | AT | CAT | 2.4 | 0 |
| Belardinelli 2008 | Both | Cardiovascular | 14 | 58.5 | 44 | 42 | cycling and treadmill | 8 | 3 | 30 | AT | CAT | 2.6 | 0.2 |
| Belardinelli 2008 | Both | Cardiovascular | 18 | 59 | 44 | 42 | dancing | 8 | 3 | 21 | AT | IAT | 2.8 | 0.2 |
| Belardinelli, 2008 | Both | Cardiovascular | 16 | 59.1 | 30 | 26 | Not specified | 8 | 3 | NR | AT | CAT | 3.2 | 0.1 |
| Benda 2016 | Both | Cardiovascular | 10 | 64.9 | 10 | 9 | cycling | 12 | 2 | 50 | AT | IAT | -0.6 | 0.1 |
| Benda 2016 | Both | Cardiovascular | 0 | 65.4 | 10 | 9 | cycling | 12 | 2 | 45 | AT | CAT | -0.5 | 0.1 |
| Berroug 2019 | Both | Cardiovascular | 39 | 66.7 | 93 | 91 | Walking | 26 | 3 | 50 | AT | CAT | -0.1 | -0.5 |
| Blumenthal 2005 | Both | Cardiovascular | 28 | 62.5 | 44 | 38 | walking, jogging | 16 | 3 | 35 | AT | CAT | 0.3 | -0.8 |
| Blumenthal 2021 | Both | Cardiovascular | 31 | 65.2 | 52 | 23 | Walking, jogging or cycling | 12 | 3 | 50 | AT | CAT | 0.5 | 0.2 |
| Boeno 2020 | Both | Cardiovascular | 60 | 45.3 | 15 | 12 | resistance training | 12 | 3 | NR | RT | DRT | 1.3 | -0.4 |
| Boeno 2020 | Both | Cardiovascular | 47 | 45.1 | 15 | 12 | treadmill | 12 | 3 | 60 | AT | CAT | 1.7 | -0.4 |
| Boff 2019 | Both | Diabetes_kidney | 61 | 23.5 | 9 | 9 | cycling | 8 | 3 | 40 | AT | IAT | 5.5 | -2.6 |
| Boff 2019 | Both | Diabetes_kidney | 50 | 22.3 | 9 | 9 | cycling | 8 | 3 | 40 | AT | CAT | 0.2 | -2.6 |
| Bouaziz 2019 | Both | Healthy | 73 | 73.6 | 27 | 29 | cycling | 9.5 | 2 | 30 | AT | IAT | 0.8 | -0.4 |
| Braith 2008 | Both | Cardiovascular | 19 | 54.4 | 9 | 7 | treadmill walking | 12 | 3 | 35 | AT | CAT | -0.4 | -2.6 |
| Briceno-Torres 2023 | male | Healthy | 0 | 20.9 | 12 | 12 | resistance training | 8 | 2 | 72 | RT | DRT | 4.4 | -0.5 |
| Briceno-Torres 2023 | male | Healthy | 0 | 20.4 | 10 | 12 | resistance training | 8 | 2 | 72 | RT | DRT | 2.7 | -0.5 |
| Casey 2007 | Both | Healthy | 55 | 21.4 | 24 | 18 | resistance training | 12 | 3 | 35 | RT | DRT | -0.1 | -0.4 |
| Choi 2023 | Women | Healthy | 100 | 39.5 | 15 | 15 | cycling, walking, resistance exercise | 12 | 7 | 55 | CT | CT | 2.6 | 0 |
| Collins 2023 | Men | Healthy | 0 | 51.1 | 15 | 14 | cycling | 12 | 3 | 55 | AT | CAT | 3.4 | 0.8 |
| Collins 2023 | Men | Healthy | 0 | 50.1 | 15 | 14 | cycling | 12 | 3 | 26 | AT | IAT | 4 | 0.8 |
| Collins 2023 | Men | Healthy | 0 | 49.2 | 15 | 14 | cycling | 12 | 3 | 26 | AT | IAT | 5 | 0.8 |
| Correia 2020 | Both | Cardiovascular | 37 | 66.6 | 29 | 50 | isometric handgrip training | 8 | 3 | NR | RT | IRT | 3.8 | 0.3 |
| Cox 2024 | Both | Diabetes_kidney | 39 | 59.2 | 23 | 23 | Treadmill running, cycling, resistance training | 8 | 3 | 26 | CT | CT | 0.6 | -0.3 |
| Cox 2024 | Both | Diabetes_kidney | 39 | 59.8 | 23 | 23 | Treadmill running, cycling, resistance training | 8 | 4 | 52.5 | CT | CT | 0.5 | -0.3 |
| Davoodi 2022 | Both | Diabetes_kidney | 42 | 53.1 | 16 | 15 | cycling | 12 | 3 | 62 | AT | IAT | 3 | 0.9 |
| Davoodi 2022 | Both | Diabetes_kidney | 45 | 54.3 | 16 | 15 | cycling | 12 | 3 | 62 | AT | CAT | 2.6 | 0.9 |
| Desch 2010 | Both | Cardiovascular | 27 | 62.3 | 14 | 12 | cycling and group exercise sessions | 25 | 7 | 60 | AT | CAT | 4.4 | -1.1 |
| Early 2020 | Both | Healthy | 37 | 23 | 10 | 10 | resistance training | 8 | 3 | NR | RT | DRT | 0.5 | -0.2 |
| Eleuteri 2013 | Male | Cardiovascular | 0 | 64.6 | 10 | 11 | cycling | 12 | 5 | 30 | AT | CAT | 1.9 | 0.5 |
| Franklin 2015 | Women | Healthy | 100 | 30.6 | 10 | 8 | resistance training | 8 | 2 | NR | RT | DRT | 0.3 | -0.4 |
| Ghardashi 2018 | Both | Diabetes_kidney | 49 | 54.5 | 18 | 17 | cycling | 12 | 3 | 60 | AT | IAT | 3.9 | 0.9 |
| Ghardashi 2018 | Both | Diabetes_kidney | 53 | 53.7 | 17 | 17 | cycling | 12 | 3 | 62 | AT | CAT | 1 | 0.9 |
| Gibbs 2012 | Both | Diabetes_kidney | 38 | 56.9 | 49 | 63 | resistance exercise, aerobic exercise | 26 | 3 | NR | CT | CT | 0.6 | -0.2 |
| Goeder 2024 | Male | Healthy | 0 | 50.9 | 23 | 12 | Cycling, resistance training | 8 | 3 | 47.5 | AT | CAT | 2.1 | -0.6 |
| Guazzi 2004 | Men | Cardiovascular | 0 | 53 | 16 | 15 | cycling | 8 | 4 | 40 | AT | CAT | 3.3 | -0.5 |
| Hansen 2023 | Both | neurological | 47 | 50 | 8 | 7 | Rowing ergometry | 12 | 3 | 30 | AT | CAT | 0.1 | -1.8 |
| Haykowsky 2009 | Both | Cardiovascular | 19 | 58 | 22 | 21 | treadmill walking and cycling and resistance training | 12 | 7 | 37.5 | CT | CT | 1.3 | 0.7 |
| Haynes 2021 | Both | Healthy | 79 | 61.9 | 17 | 16 | Land walking | 24 | 3 | 32.5 | AT | CAT | 2.4 | -0.1 |
| Haynes 2021 | Both | Healthy | 76 | 62 | 18 | 16 | Water walking | 24 | 3 | 32.5 | AT | CAT | 0.5 | -0.1 |
| He 2022 | Women | Healthy | 100 | 58 | 15 | 15 | treadmill running | 8 | 5 | 50 | AT | CAT | 0.6 | 0.1 |
| He 2022 | Women | Healthy | 100 | 46.5 | 8 | 15 | treadmill running | 8 | 3 | 40 | AT | CAT | 2.1 | 0.1 |
| He 2022 | Women | Healthy | 100 | 57.3 | 10 | 15 | treadmill running | 8 | 3 | 30 | AT | IAT | 4.6 | 0.1 |
| Herman 2011 | Both | Cardiovascular | 19 | 50.1 | 14 | 13 | cycling, staircase running | 8 | 3 | 52 | AT | IAT | 3.1 | -0.3 |
| Herrod 2019 | Women | Healthy | 100 | 35.5 | 10 | 10 | Isometric handgrip | 4 | 3 | 15 | RT | IRT | 1.1 | 0.3 |
| Hosepian 2021 | Women | Healthy | 100 | 20.5 | 13 | 13 | ergometer-based training | 10 | 4 | 40 | AT | IAT | 1.4 | -1.2 |
| Hwang 2019 | Both | Diabetes_kidney |  | 63 | 18 | 15 | Arm and leg ergometry | 8 | 4 | 40 | AT | IAT | 0.9 | -0.3 |
| Hwang 2019 | Both | Diabetes_kidney |  | 63 | 16 | 15 | Arm and leg ergometry | 8 | 4 | 47 | AT | CAT | 0.9 | -0.3 |
| Isaksen 2014 | Both | Cardiovascular | 9 | 66.3 | 24 | 11 | cycling ergometer or treadmill running | 12 | 3 | 60 | AT | IAT | 3.5 | -0.2 |
| Isaksen 2019 | Both | Cardiovascular | 7 | 67.1 | 19 | 11 | cycling or running | 12 | 3 | 60 | AT | IAT | 3.1 | -0.2 |
| Jaime 2019 | Women | Healthy | 100 | 65.2 | 12 | 8 | resistance training | 12 | NR | 27.5 | RT | DRT | -0.3 | -2 |
| Jones 2014 | Men | neoplasms | 0 | 59.5 | 25 | 25 | treadmill walking | 25 | 5 | 45 | AT | IAT | 1.7 | 0.3 |
| Kirkman 2019 | Both | Diabetes_kidney | 29 | 58.4 | 16 | 15 | cycling/jogging/elliptical trainer | 12 | 3 | 45 | AT | CAT | 1 | -1.2 |
| Kitzman 2013 | Both | Cardiovascular | 72 | 70 | 24 | 30 | Combined upper and lower body aerobic exercises | 16 | 3 | 60 | AT | CAT | -0.2 | -0.4 |
| Kobayashi 2003 | Both | Cardiovascular | 14 | 58.5 | 14 | 14 | cycling | 12 | 4 | 30 | AT | CAT | 0.2 | 0.3 |
| Kwon 2011 | Women | Diabetes_kidney | 100 | 57.3 | 13 | 15 | walking | 12 | 5 | 60 | AT | CAT | 2.1 | -0.7 |
| Kwon 2011 | Women | Diabetes_kidney | 100 | 57.7 | 12 | 15 | resistance training | 12 | 3 | 60 | RT | DRT | 0.7 | -0.7 |
| Lavrenčič 2000 | Men | Metabolic_syndrome | 0 | 52 | 14 | 15 | cycling | 12 | 3 | 50 | AT | CAT | 2 | -1.2 |
| Lee 2019 | Women | neoplasms | 100 | 46.9 | 15 | 15 | cycling | 8 | 3 | 30 | AT | IAT | 1.7 | -5.1 |
| Luk 2012 | Both | Cardiovascular | 25 | 67.2 | 32 | 32 | treadmill walking, arm ergometry, cycling, rowing, resistance exercise | 8 | 3 | 60 | CT | CT | 2.2 | 0.3 |
| McDermott 2009 | Both | Cardiovascular | 52 | 70.3 | 37 | 28 | treadmill | 24 | 3 | 15-40 | AT | CAT | -0.2 | -0.7 |
| McDermott 2009 | Both | Cardiovascular | 50 | 70.3 | 36 | 28 | resistance training | 24 | 3 | NR | RT | DRT | 1.2 | -0.7 |
| Merlo 2020 | Both | respiratory | 30 | 70 | 10 | 10 | Treadmill running or walking | 8 | 2 | 30 | AT | CAT | 3 | -0.4 |
| Molmer-Hansen 2012 | Both | Cardiovascular | 42 | 52.4 | 23 | 25 | treadmill walking/running | 12 | 3 | 47 | AT | CAT | 0.6 | 0.9 |
| Molmer-Hansen 2012 | Both | Cardiovascular | 45 | 51.9 | 25 | 25 | treadmill walking/running | 12 | 3 | 38 | AT | IAT | 4.2 | 0.9 |
| Okada 2010 | Both | Diabetes_kidney | 45 | 63.1 | 21 | 17 | dancing, cycling, resistance exercise | 12 | 4 | 75 | CT | CT | 3.6 | 1 |
| Okamoto 2007 | Both | Healthy | 68 | 18.7 | 11 | 11 | Treadmill running, resistance exercise | 8 | 2 | NR | CT | CT | -1.2 | 0.2 |
| Okamoto 2007 | Both | Healthy | 68 | 18.7 | 11 | 11 | Treadmill running , resistance exercise | 8 | 2 | NR | CT | CT | 2.3 | 0.2 |
| Okamoto 2008 | Men | Healthy | 0 | 19.4 | 10 | 9 | resistance training | 8 | 2 | NR | RT | DRT | 2.1 | 0.3 |
| Okamoto 2011 | Both | Healthy | 27 | 18.5 | 13 | 13 | resistance training | 10 | 2 | NR | RT | DRT | 2.1 | -0.1 |
| Oliveira 2019 | Both | Diabetes_kidney | 46 | 54 | 14 | 14 | cycling | 16 | 3 | 30 | AT | CAT | 2.2 | -2 |
| Olson 2006 | Women | Healthy | 100 | 38 | 15 | 15 | resistance training | 52 | 2 | NR | RT | DRT | 2.6 | -1.4 |
| Pierce 2011 | Both | Healthy | 58 | 62.2 | 26 | 10 | Walking | 8 | 4 | 45 | AT | CAT | 1.2 | 0.2 |
| Ploydang 2023 | Both | Diabetes_kidney | 64 | 69.1 | 16 | 17 | Nordic walking in water | 12 | 3 | 40 | AT | CAT | 2.4 | -0.3 |
| Prakhinkit 2014 | Women | mental | 100 | 77.9 | 13 | 13 | walking | 12 | 3 | 25 | AT | CAT | 3.8 | -0.3 |
| Pugh 2014 | Both | Metabolic_syndrome | 46 | 47.6 | 13 | 8 | ergometer-based | 16 | 3 | 38.6 | AT | CAT | 3.8 | -0.6 |
| Robinson 2016 | Both | Healthy | 70 | 31.2 | 10 | 9 | treadmill | 8 | 3 | 37.5 | AT | CAT | -0.9 | 0 |
| Sales 2020 | Both | Cardiovascular |  | 30-65 | 11 | 8 | cycling | 12 | 3 | NR | AT | IAT | 3.5 | 0 |
| Sales 2020 | Both | Cardiovascular |  | 30-65 | 11 | 8 | cycling | 12 | 3 | NR | AT | CAT | 1.8 | 0 |
| Scheer 2023 | Both | Cardiovascular | 25 | 68.9 | 14 | 11 | Water based aerobic and resistance exercise | 12 | 3 | 60 | CT | CT | 1.3 | 0.3 |
| Scheer 2023 | Both | Cardiovascular | 16 | 71 | 16 | 11 | Treadmill walking or running, cycling and resistance exercise | 12 | 3 | 60 | CT | CT | 0.1 | 0.3 |
| Shenouda 2017 | Men | Healthy | 0 | 26.6 | 9 | 6 | cycling | 12 | 3 | 10 | AT | IAT | -0.7 | 0.4 |
| Shenouda 2017 | Men | Healthy | 0 | 27.3 | 10 | 6 | cycling | 12 | 3 | 45 | AT | CAT | -1.4 | 0.4 |
| Sherwood 2016 | Both | mental | 77 | 51.1 | 51 | 49 | treadmill walking or running | 16 | 3 | 30 | AT | CAT | 0.9 | -1.5 |
| Sherwood 2016 | Both | mental | 75 | 52 | 53 | 49 | treadmill walking or running | 16 | 3 | 30 | AT | CAT | 0.5 | -1.5 |
| Sixt 2008 | Both | Cardiovascular | 26 | 64 | 13 | 10 | cycling | 4 | 7 | 60 | AT | CAT | 3.2 | 0.2 |
| Stensvold 2010 | Both | Metabolic_syndrome |  | 48.6 | 11 | 11 | treadmill | 12 | 3 | 43 | AT | IAT | 1.7 | 0.3 |
| Stensvold 2010 | Both | Metabolic_syndrome |  | 49.1 | 11 | 11 | resistance training | 12 | 3 | 45 | RT | DRT | 2.9 | 0.3 |
| Stensvold 2010 | Both | Metabolic_syndrome |  | 50 | 10 | 11 | treadmill and resistance training | 12 | 3 | 44 | CT | CT | 1.9 | 0.3 |
| Tjonna 2008 | Both | Metabolic_syndrome | 47 | 50.7 | 8 | 9 | Treadmill walking or running | 16 | 3 | 47 | AT | CAT | 4.7 | -0.8 |
| Tjonna 2008 | Both | Metabolic_syndrome | 55 | 52.7 | 11 | 9 | Treadmill walking or running | 16 | 3 | 40 | AT | IAT | 9 | -0.8 |
| Totosy 2015 | Both | neurological | 9 | 40.3 | 12 | 9 | Arm ergometry or hybrid recumbent stepper, resistance exercise | 16 | 2 | 60 | CT | CT | 1.1 | 0.2 |
| Turri-Silva 2021 | Both | Cardiovascular | 25 | 58.5 | 5 | 5 | Combined upper and lower body aerobic exercises | 12 | 3 | 50 | AT | IAT | -0.4 | 0.9 |
| Turri-Silva 2021 | Both | Cardiovascular | 21 | 55.5 | 6 | 5 | resistance training | 12 | 3 | 50 | RT | DRT | 0.4 | 0.9 |
| Twerenbold 2023 | Both | Cardiovascular | 37 | 57.5 | 19 | 19 | cycling | 8 | 3 | 45 | AT | IAT | 0.1 | 0 |
| Van 2010 | Both | Cardiovascular | 14 | 62.2 | 21 | 17 | Not reported per se | 24 | 3 | 60 | AT | CAT | 1.4 | -0.7 |
| Van 2015 | Both | Diabetes_kidney | 45 | 53.2 | 19 | 21 | cycling | 12 | 28 | 10 | AT | CAT | 0.6 | 0.1 |
| Vona 2004 | Both | Cardiovascular | 23 | 56.5 | 28 | 24 | cycling | 12 | 3 | 60 | AT | CAT | 7.7 | 2.4 |
| Vona 2009 | Both | Cardiovascular | 25 | 57 | 52 | 50 | cycling | 4 | 4 | 60 | AT | CAT | 5.4 | 0.8 |
| Vona 2009 | Both | Cardiovascular | 28 | 57.5 | 54 | 50 | resistance training | 4 | 4 | 60 | RT | DRT | 6.1 | 0.8 |
| Vona 2009 | Both | Cardiovascular | 26 | 56.5 | 53 | 50 | cycling and resistance training | 4 | 4 | 60 | CT | CT | 6.4 | 0.8 |
| Westhoff 2007 | Both | Cardiovascular | 49 | 68.7 | 24 | 27 | walking | 12 | 3 | 33 | AT | IAT | 2.3 | 0.2 |
| Westhoff 2008 | Both | Cardiovascular | 54 | 67.3 | 12 | 12 | arm-cycling | 12 | 3 | 37.5 | AT | IAT | 0.3 | 0 |
| Wislof 2007 | Both | Cardiovascular | 22 | 76 | 9 | 9 | treadmill walking | 12 | 3 | 38 | AT | IAT | 8.1 | -0.4 |
| Wislof 2007 | Both | Cardiovascular | 22 | 75 | 9 | 9 | treadmill walking | 12 | 3 | 47 | AT | CAT | 4.6 | -0.4 |
| Yoshiyawa 2010 | Women | Healthy | 100 | 57.5 | 10 | 10 | Walking or cycling | 8 | 4 | 35 | AT | CAT | 1.1 | -0.2 |

# S4 - Methodological quality assessment of individual studies

## Supplementary table 5. Fulfilment of Physiotherapy Evidence Database (PEDro) criteria for each of the studies included in the present meta-analysis

| Study name | Criterion 1 | Criterion 2 | Criterion 3 | Criterion 4 | Criterion 5 | Criterion 6 | Criterion 7 | Criterion 8 | Criterion 9 | Criterion 10 | Criterion 11 | Pedro Total |
| --- | --- | --- | --- | --- | --- | --- | --- | --- | --- | --- | --- | --- |
| Abdi et al. 2021 [1] | YES | YES | NO | YES | NO | NO | NO | YES | YES | YES | YES | 6 |
| Aispuru-Lanche et al. 2024 [2] | YES | YES | YES | YES | NO | NO | NO | YES | YES | YES | YES | 7 |
| Almenning et al. 2015 [3] | YES | YES | NO | YES | NO | NO | NO | NO | NO | YES | YES | 4 |
| Alvarez et al. 2024 [4] | YES | YES | YES | YES | YES | NO | YES | YES | NO | YES | YES | 8 |
| Azadpour et al. 2017 [5] | YES | YES | NO | YES | NO | NO | NO | YES | NO | YES | YES | 5 |
| Banks et al. 2024 [6] | YES | YES | NO | YES | NO | NO | NO | YES | NO | YES | YES | 5 |
| Beck et al. 2013 [7] | YES | YES | NO | YES | NO | NO | NO | YES | NO | YES | YES | 5 |
| Belardinelli et al. 2005 [8] | NO | YES | NO | YES | NO | NO | NO | YES | NO | YES | YES | 5 |
| Belardinelli et al. 2006 [9] | NO | YES | NO | YES | NO | NO | YES | NO | NO | YES | YES | 5 |
| Belardinelli et al. 2008 [10] | NO | YES | NO | YES | NO | NO | YES | YES | NO | YES | YES | 6 |
| Belardinelli, Lacalaprice et al. 2008 [11] | NO | YES | NO | YES | NO | NO | YES | YES | NO | YES | YES | 6 |
| Benda et al. 2015 [12] | YES | NO | NO | YES | NO | NO | NO | NO | NO | YES | YES | 3 |
| Berroug et al. 2019 [13] | YES | YES | NO | YES | NO | NO | YES | YES | NO | YES | YES | 6 |
| Blumenthal et al. 2005 [14] | YES | YES | YES | YES | NO | NO | YES | YES | YES | YES | YES | 8 |
| Blumenthal et al. 2021 [15] | YES | YES | YES | YES | NO | NO | NO | YES | YES | YES | YES | 7 |
| Boeno et al. 2020 [16] | YES | YES | NO | YES | NO | NO | YES | YES | NO | YES | YES | 6 |
| Boff et al. 2019 [17] | YES | YES | YES | YES | NO | NO | YES | NO | NO | YES | YES | 6 |
| Bouaziz et al. 2019 [18] | YES | YES | YES | YES | NO | NO | YES | YES | NO | YES | YES | 7 |
| Braith et al. 2008 [19] | NO | YES | NO | YES | NO | NO | YES | NO | NO | YES | YES | 5 |
| Briceno-Torres et al. 2023 [20] | YES | YES | NO | YES | NO | NO | NO | YES | NO | YES | YES | 5 |
| Casey et al. 2007 [21] | YES | NO | NO | YES | NO | NO | NO | YES | NO | YES | YES | 4 |
| Choi et al. 2023 [22] | YES | YES | NO | YES | NO | NO | NO | NO | NO | YES | YES | 4 |
| Collins et al. 2023 [23] | YES | YES | YES | YES | NO | NO | YES | YES | NO | YES | YES | 7 |
| Correia et al. 2020 [24] | YES | YES | YES | YES | NO | NO | YES | YES | YES | YES | YES | 8 |
| Cox et al. 2024 [25] | YES | YES | YES | NO | NO | NO | YES | YES | YES | YES | YES | 7 |
| Davoodi et al. 2022 [26] | YES | YES | NO | YES | NO | NO | YES | NO | NO | YES | YES | 5 |
| Desch et al. 2010 [27] | NO | YES | NO | YES | NO | NO | NO | YES | NO | YES | YES | 5 |
| Early et al. 2020 [28] | YES | YES | NO | YES | NO | NO | NO | NO | NO | YES | YES | 4 |
| Eleuteri et al. 2013 [29] | NO | YES | NO | YES | NO | NO | NO | YES | NO | YES | YES | 5 |
| Franklin, 2015 [30] | NO | YES | NO | YES | NO | NO | NO | YES | NO | YES | YES | 5 |
| Ghardashi Afousi et al. 2018 [31] | YES | YES | NO | YES | NO | NO | YES | NO | NO | YES | YES | 5 |
| Gibbs et al. 2012 [32] | YES | YES | NO | YES | NO | NO | NO | NO | YES | YES | YES | 5 |
| Goeder et al. 2024 [33] | YES | YES | YES | YES | NO | NO | YES | YES | NO | YES | YES | 7 |
| Guazzi et al. 2004 [34] | YES | YES | YES | YES | YES | NO | YES | YES | NO | YES | YES | 8 |
| Hansen et al. 2023 [35] | YES | YES | NO | YES | NO | NO | YES | YES | NO | YES | YES | 6 |
| Haykowsky et al. 2009 [36] | NO | YES | NO | NO | NO | NO | NO | YES | NO | YES | YES | 4 |
| Haynes et al. 2021 [37] | YES | YES | NO | YES | NO | NO | YES | YES | NO | YES | YES | 6 |
| He et al. 2022 [38] | YES | YES | NO | YES | NO | NO | YES | YES | NO | YES | YES | 6 |
| Hermann et al. 2011 [39] | YES | YES | YES | YES | NO | NO | YES | YES | NO | YES | YES | 7 |
| Herrod et al. 2019 [40] | YES | YES | NO | YES | NO | NO | NO | YES | YES | YES | YES | 6 |
| Hovsepian et al. 2021 [41] | YES | YES | YES | YES | NO | NO | YES | YES | NO | YES | YES | 7 |
| Hwang et al. 2019 [42] | YES | YES | NO | YES | NO | NO | NO | YES | NO | YES | YES | 5 |
| Isaksen et al. 2015 [43] | YES | NO | NO | YES | NO | NO | YES | YES | NO | YES | YES | 5 |
| Isaksen et al. 2019 [44] | YES | NO | NO | YES | NO | NO | YES | YES | NO | YES | YES | 5 |
| Jaime et al. 2019 [45] | NO | YES | NO | YES | NO | NO | YES | NO | NO | YES | YES | 5 |
| Jones et al. 2014 [46] | NO | YES | YES | YES | NO | NO | YES | YES | NO | YES | YES | 7 |
| Kirkman et al. 2019 [47] | YES | YES | NO | YES | NO | NO | NO | YES | NO | YES | YES | 5 |
| Kitzman et al. 2013 [48] | YES | YES | NO | YES | NO | NO | YES | YES | NO | YES | YES | 6 |
| Kobayashi et al. 2003 [49] | NO | YES | NO | YES | NO | NO | YES | YES | NO | YES | YES | 6 |
| Kwon et al. 2011 [50] | YES | YES | NO | YES | NO | NO | NO | NO | NO | YES | YES | 4 |
| Lavrenčič et al 2000 [51] | YES | YES | NO | YES | NO | NO | NO | YES | NO | NO | YES | 4 |
| Lee et al. 2019 [52] | YES | YES | NO | YES | NO | NO | NO | NO | NO | YES | YES | 4 |
| Luk et al. 2012 [53] | NO | YES | NO | YES | NO | NO | YES | YES | NO | YES | YES | 6 |
| McDermott et al. 2009 [54] | YES | YES | NO | YES | NO | NO | YES | YES | YES | YES | YES | 7 |
| Merlo et al. 2020 [55] | YES | YES | NO | YES | NO | NO | NO | YES | NO | YES | YES | 5 |
| Molmer-Hansen et al. 2012 [56] | YES | YES | NO | YES | NO | NO | NO | NO | NO | YES | YES | 4 |
| Okada et al. 2010 [57] | YES | YES | NO | YES | NO | NO | NO | YES | YES | YES | YES | 6 |
| Okamoto et al. 2007 [58] | YES | YES | NO | YES | NO | NO | YES | YES | YES | YES | YES | 7 |
| Okamoto et al. 2008 [59] | YES | YES | NO | YES | NO | NO | NO | YES | YES | YES | YES | 6 |
| Okamoto et al. 2011 [60] | YES | YES | NO | YES | NO | NO | YES | YES | YES | YES | YES | 7 |
| Oliveira e Silva 2019 [61] | NO | YES | NO | YES | NO | NO | YES | YES | YES | YES | YES | 7 |
| Olson et al. 2006 [62] | YES | YES | NO | YES | NO | NO | NO | NO | NO | YES | YES | 4 |
| Pierce et al. 2011 [63] | YES | YES | NO | YES | NO | NO | YES | NO | NO | YES | YES | 5 |
| Ploydang et al. 2023 [64] | YES | YES | NO | YES | NO | NO | NO | YES | NO | YES | YES | 5 |
| Prakhinkit et al. 2014 [65] | YES | YES | NO | YES | NO | NO | NO | YES | NO | YES | YES | 5 |
| Pugh et al. 2014 [66] | YES | YES | YES | YES | YES | NO | NO | NO | NO | YES | YES | 6 |
| Robinson et al. 2016 [67] | YES | NO | NO | YES | NO | NO | NO | NO | NO | YES | YES | 3 |
| Sales et al. 2020 [68] | YES | YES | NO | YES | NO | NO | NO | YES | NO | YES | YES | 5 |
| Scheer et al. 2023 [69] | YES | YES | YES | YES | NO | NO | YES | YES | NO | YES | YES | 7 |
| Shenouda et al. 2017 [70] | YES | NO | YES | YES | NO | NO | NO | YES | NO | YES | YES | 5 |
| Sherwood et al. 2016 [71] | YES | YES | NO | YES | NO | NO | NO | NO | YES | YES | YES | 5 |
| Sixt et al. 2008 [72] | NO | YES | NO | YES | NO | NO | YES | YES | NO | YES | NO | 5 |
| Stensvold et al. 2010 [73] | YES | YES | YES | YES | NO | NO | NO | YES | NO | YES | YES | 6 |
| Tjonna et al. 2008 [74] | NO | YES | NO | YES | NO | NO | NO | NO | NO | YES | YES | 4 |
| Totosy de Zepetnek e al. 2015 [75] | NO | YES | NO | YES | NO | NO | NO | NO | NO | YES | YES | 4 |
| Turri-Silva et al. 2021 [76] | YES | YES | YES | YES | NO | NO | YES | YES | NO | YES | YES | 7 |
| Twerenbold et al. 2023 [77] | YES | YES | NO | YES | NO | NO | YES | YES | NO | YES | YES | 6 |
| Van Craenenbroeck et al. 2015 [78] | YES | YES | YES | YES | NO | NO | YES | NO | NO | YES | YES | 6 |
| Vona et al 2009 [79] | YES | YES | NO | YES | NO | NO | YES | YES | NO | YES | YES | 6 |
| Vona et al. 2004 [80] | YES | YES | NO | YES | NO | NO | YES | YES | NO | YES | YES | 6 |
| Westhoff et al. 2007 [81] | YES | YES | NO | YES | NO | NO | YES | YES | NO | YES | YES | 6 |
| Westhoff et al. 2008 [82] | YES | YES | NO | YES | NO | NO | NO | YES | NO | YES | YES | 5 |
| Wisloff et al. 2007 [83] | YES | YES | NO | YES | NO | NO | NO | YES | NO | YES | YES | 5 |
| Yoshizawa et al. 2010 [84] | NO | YES | NO | YES | NO | NO | NO | NO | NO | YES | YES | 4 |

# S5 - Pairwise comparisons

## Supplementary table 6. Differences between healthy and symptomatic group in changes in endothelial function measured by flow-mediated dilatation technique considering following PEI in general (pairwise meta-analysis).

| Group | Effect Size | Std. Error | t | Sig. (2-tailed) | 95% Confidence Interval |  | 95% Prediction Interval |  |  | Heterogeneity Measures | Test of Subgroup Homogeneity | | |
| --- | --- | --- | --- | --- | --- | --- | --- | --- | --- | --- | --- | --- | --- |
|  |  |  |  |  | Lower | Upper | Lower | Upper | k | I-squared (%) | Omnibus (Q statistic) | k | Sig. |
| Healthy | 1.94 | 0.32 | 6.15 | p < 0.001 | 1.32 | 2.56 | -1.14 | 5.02 | 36 | 87.77 | 1.26 | 2 | 0.260 |
| Symptomatic | 2.36 | 0.21 | 11.33 | p < 0.001 | 1.95 | 2.77 | -0.76 | 5.48 | 83 | 90.5 |  |  |  |

## Supplementary table 7. Changes in endothelial function measured by flow-mediated dilatation technique considering a primary physical exercise intervention classification (pairwise meta-analysis).

| Health status | Effect Size | Std. Error | t | Sig. (2-tailed) | 95% Confidence Interval | | 95% Prediction Interval | |  | Heterogeneity Measures | Test of Subgroup Homogeneity | | | | |
| --- | --- | --- | --- | --- | --- | --- | --- | --- | --- | --- | --- | --- | --- | --- | --- |
|  |  |  |  |  | Lower | Upper | Lower | Upper | k | I-squared (%) | Omnibus (Q statistic) | | k | | Sig. |
| AT | 2.36 | 0.22 | 10.92 | p < 0.001 | 1.94 | 2.79 | -0.89 | 5.62 | 82 | 91.7 |  | | | | |
| CT | 1.65 | 0.47 | 3.5 | p < 0.001 | 0.73 | 2.58 | -1.58 | 4.89 | 17 | 81.1 |  |  |  |  |  |
| RT | 2.25 | 0.37 | 6.11 | p < 0.001 | 1.53 | 2.97 | -0.32 | 4.82 | 20 | 77.4 | 1.85 | 3 | | 0.396 | |
| AT, aerobic training; RT, resistance training; and CT, combined training (CT=AE + RT). | | | | | | | | | | | | | | | |

## Supplementary table 8. Changes in endothelial function measured by flow-mediated dilatation technique considering a secondary physical exercise intervention classification (pairwise meta-analysis).

|  | Effect Size | Std. Error | t | Sig. (2-tailed) | 95% Confidence Interval | | 95% Prediction Interval | |  | Heterogeneity Measures | Test of Subgroup Homogeneity | | |
| --- | --- | --- | --- | --- | --- | --- | --- | --- | --- | --- | --- | --- | --- |
|  |  |  |  |  | Lower | Upper | Lower | Upper | k | I-squared (%) | Omnibus (Q statistic) | k | Sig. |
| CAT | 2.12 | 0.23 | 9.04 | p < 0.001 | 1.66 | 2.58 | -0.62 | 4.87 | 52 | 90.3 |  | | |
| CT | 1.65 | 0.47 | 3.5 | p < 0.001 | 0.73 | 2.58 | -1.58 | 4.89 | 17 | 81.1 |  |  |  |
| DRT | 2.25 | 0.39 | 5.75 | p < 0.001 | 1.48 | 3.01 | -0.39 | 4.89 | 18 | 79.2 |  |  |  |
| IAT | 2.91 | 0.46 | 6.36 | p < 0.001 | 2.01 | 3.81 | -1.51 | 7.33 | 30 | 91.6 | 3.86 | 4 | 0.277 |
| CAT, continuous aerobic training (AT); IAT, interval AT; DRT, dynamic resistance training (RT); and CT, combined training (CT=AE + RT). | | | | | | | | | | | | | |

## Supplementary table 9. Changes in endothelial function measured by flow-mediated dilatation technique considering a intervention duration (pairwise meta-analysis).

|  | Effect Size | Std. Error | t | Sig. (2-tailed) | 95% Confidence Interval | | 95% Prediction Intervalb | |  | Heterogeneity Measures | Test of Subgroup Homogeneity | | | |
| --- | --- | --- | --- | --- | --- | --- | --- | --- | --- | --- | --- | --- | --- | --- |
|  |  |  |  |  | Lower | Upper | Lower | Upper | k | I-squared (%) | Omnibus (Q statistic) | k | | Sig. |
| <=4 weeks | 4.28 | 0.77 | 5.54 | p < 0.001 | 2.77 | 5.79 | 1.04 | 7.52 | 5 | 82.2 |  | | | |
| 5 to 12 weeks | 2.07 | 0.18 | 11.47 | p < 0.001 | 1.72 | 2.43 | -0.69 | 4.84 | 94 | 86.9 |  |  |  |  |
| 13 to 24 weeks | 2.65 | 0.61 | 4.37 | p < 0.001 | 1.46 | 3.84 | -1.56 | 6.86 | 15 | 95.0 |  |  |  |  |
| 25+ weeks | 1.77 | 0.75 | 2.38 | 0.017 | 0.31 | 3.23 | -1.28 | 4.83 | 5 | 79.3 | 8.60 | 4 | 0.035 | |

# S6 - Credibility assessment

## Supplementary table 10. Level of concern for each of the six domains for each comparison in the Network Meta-analysis within primary classification of PEI


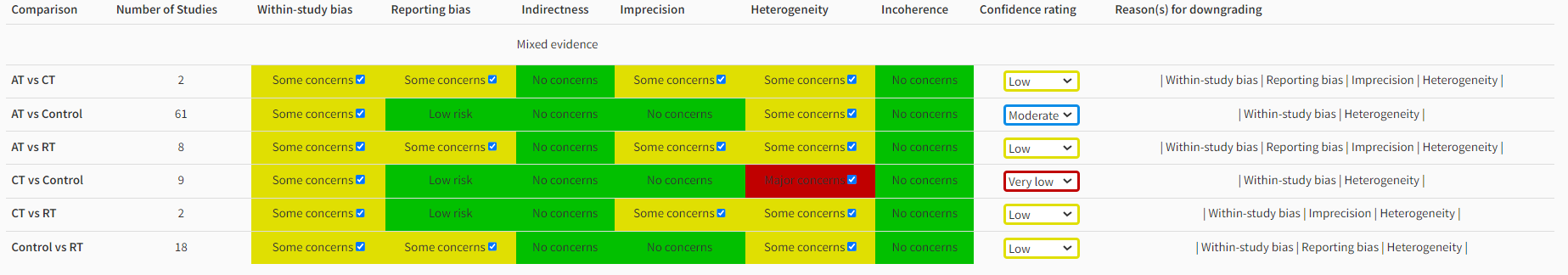

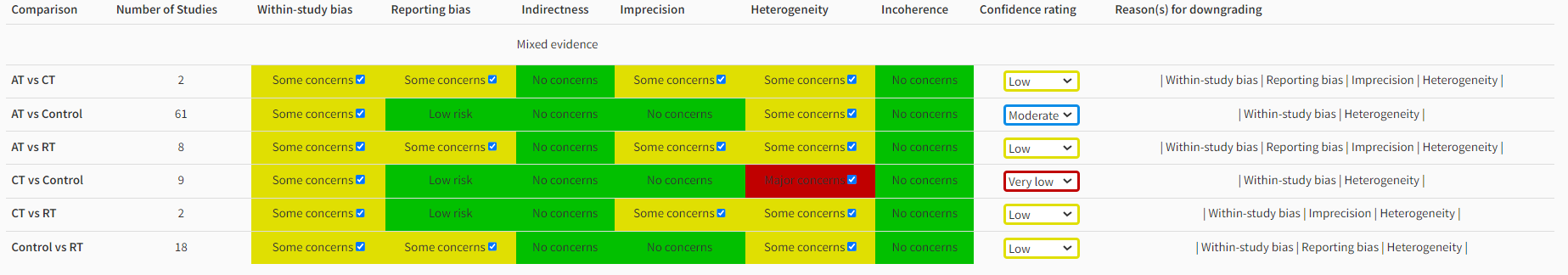


# References

1. Abdi S, Tadibi V, Sheikholeslami-Vatani D. Effect of High-intensity Interval Training on Endothelial Function in Type 2 Diabetic Females. Asian J Sports Med [Internet]. 2021;12:1–6. Available from: https://search.ebscohost.com/login.aspx?direct=true&AuthType=shib,ip,url,uid&db=cin20&AN=154409469&lang=it&site=ehost-live&custid=s3680714

2. Aispuru-lanche R, Jayo-montoya JA, Maldonado-martín S. Vascular-endothelial adaptations following low and high volumes of high-intensity interval training in patients after myocardial infarction. 2024;1–18.

3. Almenning I, Rieber-Mohn A, Lundgren KM, Shetelig Løvvik T, Garnæs KK, Moholdt T. Effects of High Intensity Interval Training and Strength Training on Metabolic, Cardiovascular and Hormonal Outcomes in Women with Polycystic Ovary Syndrome: A Pilot Study. Lambalk CB, editor. PLoS One [Internet]. 2015;10:e0138793. Available from: https://dx.plos.org/10.1371/journal.pone.0138793

4. Alvarez C, Peñailillo L, Ibacache-Saavedra P, Jerez-Mayorga D, Campos-Jara C, Andrade DC, et al. Six weeks of a concurrent training therapy improves endothelial function and arterial stiffness in hypertensive adults with minimum non-responders. Hipertens y Riesgo Vasc. 2024;

5. Azadpour N, Tartibian B, Koşar ŞN. Effects of aerobic exercise training on ACE and ADRB2 gene expression, plasma angiotensin II level, and flow-mediated dilation: a study on obese postmenopausal women with prehypertension. Menopause [Internet]. 2017;24:269–77. Available from: https://journals.lww.com/00042192-201703000-00007

6. Banks NF, Rogers EM, Jenkins NDM, Stanhewicz AE, Whitaker KM. Resistance exercise lowers blood pressure and improves vascular endothelial function in individuals with elevated blood pressure or stage-1 hypertension. 2025;

7. Beck DT, Casey DP, Martin JS, Emerson BD, Braith RW. Exercise training improves endothelial function in young prehypertensives. Exp Biol Med. 2013;238:433–41.

8. Belardinelli R, Lacalaprice F, Faccenda E, Purcaro A, Perna G. Effects of short-term moderate exercise training on sexual function in male patients with chronic stable heart failure. Int J Cardiol. 2005;101:83–90.

9. Belardinelli R, Capestro F, Misiani A, Scipione P, Georgiou D. Moderate exercise training improves functional capacity, quality of life, and endothelium-dependent vasodilation in chronic heart failure patients with implantable cardioverter defibrillators and cardiac resynchronization therapy. Eur J Prev Cardiol. 2006;13:818–25.

10. Belardinelli R, Lacalaprice F, Faccenda E, Volpe L. Trimetazidine potentiates the effects of exercise training in patients with ischemic cardiomyopathy referred for cardiac rehabilitation. Eur J Cardiovasc Prev Rehabil. 2008;15:533–40.

11. Belardinelli R, Lacalaprice F, Ventrella C, Volpe L, Faccenda E. Waltz Dancing in Patients With Chronic Heart Failure. Circ Hear Fail [Internet]. 2008;1:107–14. Available from: https://www.ahajournals.org/doi/10.1161/CIRCHEARTFAILURE.108.765727

12. Benda NMM, Seeger JPH, Stevens GGCF, Hijmans-Kersten BTP, van Dijk APJ, Bellersen L, et al. Effects of High-Intensity Interval Training versus Continuous Training on Physical Fitness, Cardiovascular Function and Quality of Life in Heart Failure Patients. Hosoda T, editor. PLoS One [Internet]. 2015;10:e0141256. Available from: https://dx.plos.org/10.1371/journal.pone.0141256

13. Berroug J, Korcarz CE, Mitchell CKC, Weber JM, Tian L, McDermott MM, et al. Brachial artery intima-media thickness and grayscale texture changes in patients with peripheral artery disease receiving supervised exercise training in the PROPEL randomized clinical trial. Vasc Med. 2019;24:12–22.

14. Blumenthal JA, Sherwood A, Babyak MA, Watkins LL, Waugh R, Georgiades A, et al. Effects of Exercise and Stress Management Training on Markers of Cardiovascular Risk in Patients With Ischemic Heart Disease. JAMA [Internet]. 2005;293:1626. Available from: http://jama.jamanetwork.com/article.aspx?doi=10.1001/jama.293.13.1626

15. Blumenthal JA, Smith PJ, Jiang W, Hinderliter A, Watkins LL, Hoffman BM, et al. Effect of Exercise, Escitalopram, or Placebo on Anxiety in Patients with Coronary Heart Disease: The Understanding the Benefits of Exercise and Escitalopram in Anxious Patients with Coronary Heart Disease (UNWIND) Randomized Clinical Trial. JAMA Psychiatry. 2021;78:1270–8.

16. Boeno FP, Ramis TR, Munhoz S V., Farinha JB, Moritz CEJ, Leal-Menezes R, et al. Effect of aerobic and resistance exercise training on inflammation, endothelial function and ambulatory blood pressure in middle-aged hypertensive patients. J Hypertens. 2020;38:2501–9.

17. Boff W, da Silva AM, Farinha JB, Rodrigues-Krause J, Reischak-Oliveira A, Tschiedel B, et al. Superior Effects of High-Intensity Interval vs. Moderate-Intensity Continuous Training on Endothelial Function and Cardiorespiratory Fitness in Patients With Type 1 Diabetes: A Randomized Controlled Trial. Front Physiol. 2019;10.

18. Bouaziz W, Lang P-O, Schmitt E, Leprêtre P-M, Lefebvre F, Momas C, et al. Effects of a short-term interval aerobic training program with recovery bouts on vascular function in sedentary aged 70 or over: A randomized controlled trial. Arch Gerontol Geriatr [Internet]. 2019;82:217–25. Available from: https://linkinghub.elsevier.com/retrieve/pii/S016749431930055X

19. Braith RW, Schofield RS, Hill JA, Casey DP, Pierce GL. Exercise Training Attenuates Progressive Decline in Brachial Artery Reactivity in Heart Transplant Recipients. J Hear Lung Transplant. 2008;27:52–9.

20. Briceño-Torres JM, Carpio-Rivera E, Solera-Herrera A, Forsse J, Grandjean PW, Moncada-Jiménez J. Low-Intensity Resistance Training Improves Flow-Mediated Dilation in Young Hispanic Adults. J Strength Cond Res. 2023;37:298–304.

21. Casey DP, Beck DT, Braith RW. Progressive resistance training without volume increases does not alter arterial stiffness and aortic wave reflection. Exp Biol Med. 2007;232:1228–35.

22. Choi J-H, Kim S-W, Seo J, Sun Y, Jung W-S, Park H-Y, et al. Effects of a Mobile-Health Exercise Intervention on Body Composition, Vascular Function, and Autonomic Nervous System Function in Obese Women: A Randomized Controlled Trial. J Multidiscip Healthc [Internet]. 2023;Volume 16:1601–15. Available from: https://www.dovepress.com/effects-of-a-mobile-health-exercise-intervention-on-body-composition-v-peer-reviewed-fulltext-article-JMDH

23. Collins BEG, Donges C, Robergs R, Cooper J, Sweeney K, Kingsley M. Moderate continuous- and high-intensity interval training elicit comparable cardiovascular effect among middle-aged men regardless of recovery mode. Eur J Sport Sci. 2023;

24. A. Correia M, Oliveira PL, Farah BQ, Vianna LC, Wolosker N, Puech‐Leao P, et al. Effects of Isometric Handgrip Training in Patients With Peripheral Artery Disease: A Randomized Controlled Trial. J Am Heart Assoc [Internet]. 2020;9. Available from: https://www.ahajournals.org/doi/10.1161/JAHA.119.013596

25. Cox ER, Gajanand T, Keating SE, Hordern MD, Burton NW, Green DJ, et al. Effect of low-volume combined aerobic and resistance high-intensity interval training on vascular health in people with type 2 diabetes: a randomised controlled trial. Eur J Appl Physiol. 2024;124:2819–33.

26. Davoodi M, Hesamabadi BK, Ariabood E, Izadi MR, Ghardashi‐Afousi A, Bigi MAB, et al. Improved blood pressure and flow‐mediated dilatation via increased plasma adropin and nitrate/nitrite induced by high‐intensity interval training in patients with type 2 diabetes. Exp Physiol [Internet]. 2022;107:813–24. Available from: https://physoc.onlinelibrary.wiley.com/doi/10.1113/EP089371

27. Desch S, Sonnabend M, Niebauer J, Sixt S, Sareban M, Eitel I, et al. Effects of physical exercise versus rosiglitazone on endothelial function in coronary artery disease patients with prediabetes. DIABETES Obes Metab. 2010;12:825–8.

28. Early KS, Rockhill M, Bryan A, Tyo B, Buuck D, McGinty J. EFFECT OF BLOOD FLOW RESTRICTION TRAINING ON MUSCULAR PERFORMANCE, PAIN AND VASCULAR FUNCTION. Int J Sports Phys Ther [Internet]. 2020;15:892–900. Available from: https://spts.org/member-benefits-detail/enjoy-member-benefits/journals/ijspt/v15n6#ijspt20200892

29. Eleuteri E, Mezzani A, Di Stefano A, Vallese D, Gnemmi I, Delle Donne L, et al. Aerobic training and angiogenesis activation in patients with stable chronic heart failure: a preliminary report. Biomarkers [Internet]. 2013;18:418–24. Available from: https://revistas.ufrj.br/index.php/rce/article/download/1659/1508%0Ahttp://hipatiapress.com/hpjournals/index.php/qre/article/view/1348%5Cnhttp://www.tandfonline.com/doi/abs/10.1080/09500799708666915%5Cnhttps://mckinseyonsociety.com/downloads/reports/Educa

30. Franklin NC, Robinson AT, Bian J-T, Ali MM, Norkeviciute E, McGinty P, et al. Circuit Resistance Training Attenuates Acute Exertion-Induced Reductions in Arterial Function but Not Inflammation in Obese Women. Metab Syndr Relat Disord [Internet]. 2015;13:227–34. Available from: http://www.liebertpub.com/doi/10.1089/met.2014.0135

31. Ghardashi Afousi A, Izadi MR, Rakhshan K, Mafi F, Biglari S, Gandomkar Bagheri H. Improved brachial artery shear patterns and increased flow‐mediated dilatation after low‐volume high‐intensity interval training in type 2 diabetes. Exp Physiol [Internet]. 2018;103:1264–76. Available from: https://physoc.onlinelibrary.wiley.com/doi/10.1113/EP087005

32. Barone Gibbs B, Dobrosielski DA, Bonekamp S, Stewart KJ, Clark JM. A randomized trial of exercise for blood pressure reduction in type 2 diabetes: Effect on flow-mediated dilation and circulating biomarkers of endothelial function. Atherosclerosis. 2012;224:446–53.

33. Goeder D, Kröpfl JM, Angst T, Hanssen H, Hauser C, Infanger D, et al. VascuFit: Aerobic exercise improves endothelial function independent of cardiovascular risk: A randomized-controlled trial. Atherosclerosis [Internet]. 2024;399:118631. Available from: https://www.atherosclerosis-journal.com/article/S0021-9150(24)01203-6/fulltext

34. Guazzi M, Reina G, Tumminello G, Guazzi MD. Improvement of alveolar-capillary membrane diffusing capacity with exercise training in chronic heart failure. J Appl Physiol [Internet]. 2004;97:1866–73. Available from: https://www.physiology.org/doi/10.1152/japplphysiol.00365.2004

35. Hansen RK, Samani A, Laessoe U, Handberg A, Mellergaard M, Figlewski K, et al. Rowing exercise increases cardiorespiratory fitness and brachial artery diameter but not traditional cardiometabolic risk factors in spinal cord-injured humans. Eur J Appl Physiol [Internet]. 2023;123:1241–55. Available from: https://link.springer.com/10.1007/s00421-023-05146-y

36. Haykowsky M, Taylor D, Kim D, Tymchak W. Exercise training improves aerobic capacity and skeletal muscle function in heart transplant recipients. Am J Transplant [Internet]. 2009;9:734–9. Available from: https://doi.org/10.1111/j.1600-6143.2008.02531.x

37. Haynes A, Naylor LH, Spence AL, Robey E, Cox KL, Maslen BA, et al. Effects of Land versus Water Walking Interventions on Vascular Function in Older Adults. Med Sci Sports Exerc. 2021;53:83–9.

38. He H, Wang C, Chen X, Sun X, Wang Y, Yang J, et al. The effects of HIIT compared to MICT on endothelial function and hemodynamics in postmenopausal females. J Sci Med Sport [Internet]. 2022;25:364–71. Available from: https://doi.org/10.1016/j.jsams.2022.01.007

39. Hermann TS, Dall CH, Christensen SB, Goetze JP, Prescott E, Gustafsson F. Effect of High Intensity Exercise on Peak Oxygen Uptake and Endothelial Function in Long-Term Heart Transplant Recipients. Am J Transplant. 2011;11:536–41.

40. Herrod PJJ, Blackwell JEM, Moss BF, Gates A, Atherton PJ, Lund JN, et al. The efficacy of ‘static’ training interventions for improving indices of cardiorespiratory fitness in premenopausal females. Eur J Appl Physiol [Internet]. 2019;119:645–52. Available from: http://link.springer.com/10.1007/s00421-018-4054-1

41. Hovsepian V, Marandi SM, Esfarjani F, Zavar R, Sadeghi M. The Effect of All Extremity High Intensity Interval Training on Athero-Protective Factors and Endothelial Function in Overweight and Obese Women. Int J Prev Med [Internet]. 2021;12:1–6. Available from: http://10.0.16.7/ijpvm.IJPVM_248_19

42. Hwang CL, Lim J, Yoo JK, Kim HK, Hwang MH, Handberg EM, et al. High-Intensity Interval Training and Moderate-Intensity Continuous Training Improve Endothelial Function Similarly in Adults with Type 2 Diabetes. FASEB J. 2019;33 MA-5.

43. Isaksen K, Munk PS, Valborgland T, Larsen AI. Aerobic interval training in patients with heart failure and an implantable cardioverter defibrillator: a controlled study evaluating feasibility and effect. Eur J Prev Cardiol. 2015;22:296–303.

44. Isaksen K, Halvorsen B, Munk PS, Aukrust P, Larsen AI. Effects of interval training on inflammatory biomarkers in patients with ischemic heart failure. Scand Cardiovasc J. 2019;53:213–9.

45. Jaime SJ, Maharaj A, Alvarez-Alvarado S, Figueroa A. Impact of low-intensity resistance and whole-body vibration training on aortic hemodynamics and vascular function in postmenopausal women. Hypertens Res [Internet]. 2019;42:1979–88. Available from: https://www.nature.com/articles/s41440-019-0328-1

46. Jones LW, Hornsby WE, Freedland SJ, Lane A, West MJ, Moul JW, et al. Effects of Nonlinear Aerobic Training on Erectile Dysfunction and Cardiovascular Function Following Radical Prostatectomy for Clinically Localized Prostate Cancer. Eur Urol. 2014;65:852–5.

47. Kirkman DL, Ramick MG, Muth BJ, Stock JM, Pohlig RT, Townsend RR, et al. Effects of aerobic exercise on vascular function in nondialysis chronic kidney disease: a randomized controlled trial. Am J Physiol Physiol. 2019;316:F898–905.

48. Kitzman DW, Brubaker PH, Herrington DM, Morgan TM, Stewart KP, Hundley WG, et al. Effect of Endurance Exercise Training on Endothelial Function and Arterial Stiffness in Older Patients With Heart Failure and Preserved Ejection Fraction. J Am Coll Cardiol [Internet]. 2013;62:584–92. Available from: https://linkinghub.elsevier.com/retrieve/pii/S0735109713017956

49. Kobayashi N, Tsuruya Y, Iwasawa T, Ikeda N, Hashimoto S, Yasu T, et al. Exercise Training in Patients With Chronic Heart Failure Improves Endothelial Function Predominantly in the Trained Extremities. Circ J [Internet]. 2003;67:505–10. Available from: http://www.jstage.jst.go.jp/article/circj/67/6/67_6_505/_article

50. Kwon HR, Min KW, Ahn HJ, Seok HG, Lee JH, Park GS, et al. Effects of Aerobic Exercise vs. Resistance Training on Endothelial Function in Women with Type 2 Diabetes Mellitus. Diabetes Metab J [Internet]. 2011;35:364. Available from: http://e-dmj.org/journal/view.php?doi=10.4093/dmj.2011.35.4.364

51. Lavrenčič A, Salobir BG, Keber I. Physical training improves flow-mediated dilation in patients with the polymetabolic syndrome. Arterioscler Thromb Vasc Biol. 2000;20:551–5.

52. Lee K, Kang I, Mack WJ, Mortimer J, Sattler F, Salem G, et al. Effects of high-intensity interval training on vascular endothelial function and vascular wall thickness in breast cancer patients receiving anthracycline-based chemotherapy: a randomized pilot study. Breast Cancer Res Treat [Internet]. 2019;177:477–85. Available from: https://link.springer.com/10.1007/s10549-019-05332-7

53. Luk TH, Dai YL, Siu CW, Yiu KH, Chan HT, Lee SWL, et al. Effect of exercise training on vascular endothelial function in patients with stable coronary artery disease: a randomized controlled trial. Eur J Prev Cardiol. 2012;19:830–9.

54. McDermott MM, Ades P, Guralnik JM, Dyer A, Ferrucci L, Liu K, et al. Treadmill Exercise and Resistance Training in Patients With Peripheral Arterial Disease With and Without Intermittent Claudication. JAMA [Internet]. 2009;301:165. Available from: http://jama.jamanetwork.com/article.aspx?doi=10.1001/jama.2008.962

55. Merlo C, Bernardi E, Bellotti F, Pomidori L, Cogo A. Supervised exercise training improves endothelial function in COPD patients: a method to reduce cardiovascular risk? ERJ OPEN Res. 2020;6.

56. Molmen-Hansen HE, Stolen T, Tjonna AE, Aamot IL, Ekeberg IS, Tyldum GA, et al. Aerobic interval training reduces blood pressure and improves myocardial function in hypertensive patients. Eur J Prev Cardiol. 2012;19:151–60.

57. Okada S, Hiuge A, Makino H, Nagumo A, Takaki H, Konishi H, et al. Effect of Exercise Intervention on Endothelial Function and Incidence of Cardiovascular Disease in Patients with Type 2 Diabetes. J Atheroscler Thromb. 2010;17:828–33.

58. Okamoto T, Masuhara M, Ikuta K. Combined aerobic and resistance training and vascular function: effect of aerobic exercise before and after resistance training. J Appl Physiol. 2007;103:1655–61.

59. Okamoto T, Masuhara M, Ikuta K. Effects of low-intensity resistance training with slow lifting and lowering on vascular function. J Hum Hypertens [Internet]. 2008;22:509–11. Available from: https://www.nature.com/articles/jhh200812

60. Okamoto T, Masuhara M, Ikuta K. Effect of low-intensity resistance training on arterial function. Eur J Appl Physiol [Internet]. 2011;111:743–8. Available from: http://link.springer.com/10.1007/s00421-010-1702-5

61. Oliveira E Silva VR, Stringuetta Belik F, Hueb JC, De Souza Gonçalves R, Costa Teixeira Caramori J, Perez Vogt B, et al. Aerobic Exercise Training and Nontraditional Cardiovascular Risk Factors in Hemodialysis Patients: Results from a Prospective Randomized Trial. CardioRenal Med. 2019;9:391–9.

62. OLSON TP, DENGEL DR, LEON AS, SCHMITZ KH. Moderate Resistance Training and Vascular Health in Overweight Women. Med Sci Sport Exerc [Internet]. 2006;38:1558–64. Available from: https://journals.lww.com/00005768-200609000-00004

63. Pierce GL, Eskurza I, Walker AE, Fay TN, Seals DR. Sex-specific effects of habitual aerobic exercise on brachial artery flow-mediated dilation in middle-aged and older adults. Clin Sci [Internet]. 2011;120:13–23. Available from: https://portlandpress.com/clinsci/article/120/1/13/68781/Sex-specific-effects-of-habitual-aerobic-exercise

64. Ploydang T, Khovidhunkit W, Tanaka H, Suksom D. Nordic Walking in Water on Cerebrovascular Reactivity and Cognitive Function in Elderly Patients with Type 2 Diabetes. Med Sci Sports Exerc. 2023;55:1803–11.

65. Prakhinkit S, Suppapitiporn S, Tanaka H, Suksom D. Effects of buddhism walking meditation on depression, functional fitness, and endothelium-dependent vasodilation in depressed elderly. J Altern Complement Med. 2014;20:411–6.

66. Pugh CJA, Sprung VS, Kemp GJ, Richardson P, Shojaee-Moradie F, Margot Umpleby A, et al. Exercise training reverses endothelial dysfunction in nonalcoholic fatty liver disease. Am J Physiol - Hear Circ Physiol. 2014;307:H1298–306.

67. Robinson AT, Franklin NC, Norkeviciute E, Bian JT, Babana JC, Szczurek MR, et al. Improved arterial flow-mediated dilation after exertion involves hydrogen peroxide in overweight and obese adults following aerobic exercise training. J Hypertens [Internet]. 2016;34:1309–16. Available from: https://journals.lww.com/00004872-201607000-00012

68. Sales ARK, Azevedo LF, Silva TOC, Rodrigues AG, Oliveira PA, Jordão CP, et al. High-Intensity Interval Training Decreases Muscle Sympathetic Nerve Activity and Improves Peripheral Vascular Function in Patients With Heart Failure With Reduced Ejection Fraction. Circ Hear Fail [Internet]. 2020;13. Available from: https://www.ahajournals.org/doi/10.1161/CIRCHEARTFAILURE.120.007121

69. Scheer AS, Oliveira BIR De, Shah A, Green DJ, Maiorana AJ, Jacques A, et al. The effects of water-based circuit exercise training on vascular function in people with coronary heart disease. 2025;

70. Shenouda N, Gillen JB, Gibala MJ, MacDonald MJ. Changes in brachial artery endothelial function and resting diameter with moderate-intensity continuous but not sprint interval training in sedentary men. J Appl Physiol [Internet]. 2017;123:773–80. Available from: https://www.physiology.org/doi/10.1152/japplphysiol.00058.2017

71. Sherwood A, Blumenthal JA, Smith PJ, Watkins LL, Hoffman BM, Hinderliter AL. Effects of Exercise and Sertraline on Measures of Coronary Heart Disease Risk in Patients With Major Depression: Results From the SMILE-II Randomized Clinical Trial. Psychosom Med [Internet]. 2016;78:602–9. Available from: file:///C:/Users/Carla Carolina/Desktop/Artigos para acrescentar na qualificação/The impact of birth weight on cardiovascular disease risk in the.pdf

72. Sixt S, Rastan A, Desch S, Sonnabend M, Schmidt A, Schuler G, et al. Exercise training but not rosiglitazone improves endothelial function in prediabetic patients with coronary disease. Eur J Cardiovasc Prev Rehabil. 2008;15:473–8.

73. Stensvold D, Tjønna AE, Skaug EA, Aspenes S, Stølen T, Wisløff U, et al. Strength training versus aerobic interval training to modify risk factors of metabolic syndrome. J Appl Physiol. 2010;108:804–10.

74. Tjønna AE, Lee SJ, Rognmo Ø, Stølen TO, Bye A, Haram PM, et al. Aerobic interval training versus continuous moderate exercise as a treatment for the metabolic syndrome: a pilot study. Circulation [Internet]. 2008;118:346–54. Available from: http://www.ncbi.nlm.nih.gov/pubmed/18606913

75. Totosy de Zepetnek JO, Pelletier CA, Hicks AL, MacDonald MJ. Following the Physical Activity Guidelines for Adults With Spinal Cord Injury for 16 Weeks Does Not Improve Vascular Health: A Randomized Controlled Trial. Arch. Phys. Med. Rehabil. 2015. p. 1566–75.

76. Turri-Silva N, Vale-Lira A, Verboven K, Quaglioti Durigan JL, Hansen D, Cipriano G. High-intensity interval training versus progressive high-intensity circuit resistance training on endothelial function and cardiorespiratory fitness in heart failure: A preliminary randomized controlled trial. Abdelbasset WK, editor. PLoS One [Internet]. 2021;16:e0257607. Available from: https://dx.plos.org/10.1371/journal.pone.0257607

77. Twerenbold S, Hauser C, Gander J, Carrard J, Gugleta K, Hinrichs T, et al. Short-term high-intensity interval training improves micro- but not macrovascular function in hypertensive patients. Scand J Med Sci Sports. 2023;

78. Van Craenenbroeck AH, Van Craenenbroeck EM, Van Ackeren K, Vrints CJ, Conraads VM, Verpooten GA, et al. Effect of Moderate Aerobic Exercise Training on Endothelial Function and Arterial Stiffness in CKD Stages 3-4: A Randomized Controlled Trial. Am J Kidney Dis [Internet]. 2015;66:285–96. Available from: http://dx.doi.org/10.1053/j.ajkd.2015.03.015

79. Vona M, Codeluppi GM, Iannino T, Ferrari E, Bogousslavsky J, von Segesser LK. Effects of Different Types of Exercise Training Followed by Detraining on Endothelium-Dependent Dilation in Patients With Recent Myocardial Infarction. Circulation [Internet]. 2009;119:1601–8. Available from: https://www.ahajournals.org/doi/10.1161/CIRCULATIONAHA.108.821736

80. Vona M, Rossi A, Capodaglio P, Rizzo S, Servi P, De Marchi M, et al. Impact of physical training and detraining on endothelium-dependent vasodilation in patients with recent acute myocardial infarction. Am Heart J [Internet]. 2004;147:1039–46. Available from: https://linkinghub.elsevier.com/retrieve/pii/S0002870304000146

81. Westhoff TH, Franke N, Schmidt S, Vallbracht-Israng K, Zidek W, Dimeo F, et al. Beta-blockers do not impair the cardiovascular benefits of endurance training in hypertensives. J Hum Hypertens. 2007;21:486–93.

82. Westhoff TH, Schmidt S, Gross V, Joppke M, Zidek W, van der Giet M, et al. The cardiovascular effects of upper-limb aerobic exercise in hypertensive patients. J Hypertens. 2008;26:1336–42.

83. Wisløff U, Støylen A, Loennechen JP, Bruvold M, Rognmo Ø, Haram PM, et al. Superior Cardiovascular Effect of Aerobic Interval Training Versus Moderate Continuous Training in Heart Failure Patients. Circulation [Internet]. 2007;115:3086–94. Available from: https://www.ahajournals.org/doi/10.1161/CIRCULATIONAHA.106.675041

84. Yoshizawa M, Maeda S, Miyaki A, Misono M, Choi Y, Shimojo N, et al. Additive Beneficial Effects of Lactotripeptides Intake With Regular Exercise on Endothelium-Dependent Dilatation in Postmenopausal Women. Am J Hypertens [Internet]. 2010;23:368–72. Available from: https://academic.oup.com/ajh/article-lookup/doi/10.1038/ajh.2009.270
